# Linker-Functionalized Phosphinate Metal–Organic Frameworks: Adsorbents for the Removal of Emerging Pollutants

**DOI:** 10.1021/acs.inorgchem.3c01810

**Published:** 2023-09-08

**Authors:** Soňa Ondrušová, Daniel Bůžek, Matouš Kloda, Jan Rohlíček, Slavomír Adamec, Miroslav Pospíšil, Pavel Janoš, Jan Demel, Jan Hynek

**Affiliations:** † Institute of Inorganic Chemistry of the Czech Academy of Sciences, Husinec-Řež 1001, Řež 250 68, Czech Republic; ‡ Department of Inorganic Chemistry, Faculty of Science, Charles University, Hlavova 2030, Prague 128 40, Czech Republic; § Department of Environmental Chemistry and Technology, Faculty of Environment, Jan Evangelista Purkyně University in Ústí nad Labem, Pasteurova 3632/15, Ústí nad Labem 400 96, Czech Republic; ∥ Institute of Physics of the Czech Academy of Sciences, Na Slovance 1999/2, Prague 182 21, Czech Republic; ⊥ Department of Chemical Physics and Optics, Faculty of Mathematics and Physics, Charles University, Ke Karlovu 3, Prague 121 16, Czech Republic

## Abstract

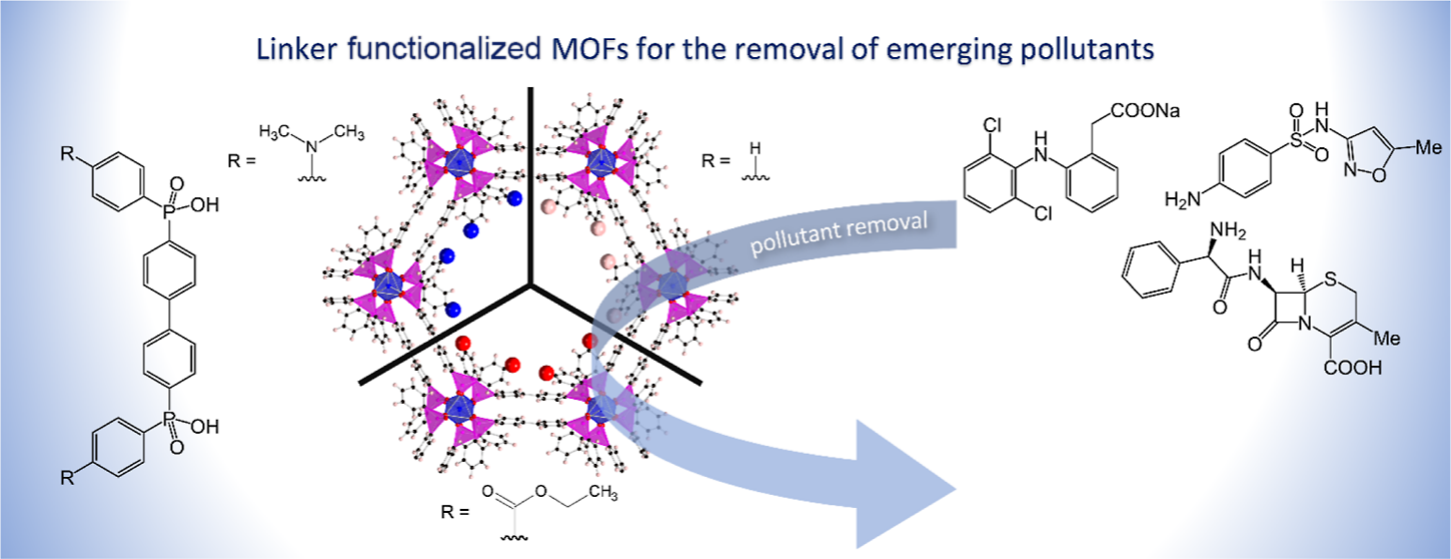

Metal–organic frameworks (MOFs) are attracting increasing attention as adsorbents of contaminants of emerging concern that are difficult to remove by conventional processes. This paper examines how functional groups covering the pore walls of phosphinate-based MOFs affect the adsorption of specific pharmaceutical pollutants (diclofenac, cephalexin, and sulfamethoxazole) and their hydrolytic stability. New structures, isoreticular to the phosphinate MOF ICR-7, are presented. The phenyl ring facing the pore wall of the presented MOFs is modified with dimethylamino groups (ICR-8) and ethyl carboxylate groups (ICR-14). These functionalized MOFs were obtained from two newly synthesized phosphinate linkers containing the respective functional groups. The presence of additional functional groups resulted in higher affinity toward the tested pollutants compared to ICR-7 or activated carbon. However, this modification also comes with a reduced adsorption capacity. Importantly, the introduction of the functional groups enhanced the hydrolytic stability of the MOFs.

## Abstract 2

The graphical abstract depicts the formation of the ICR MOFs with various linkers containing the biphenylene spacer. The synthesis starts from H_2_BBP(Ph) and its derivatives bearing −NMe_2_ and −COOEt functional groups. In the shown structure, the blue balls represent −NMe_2_ groups, the red balls stand for −COOEt, and the beige balls are used in the case of the non-functionalized linker. The right part of the graphical abstract depicts the effective removal of three different pharmaceutical pollutants—diclofenac, cephalexin, and sulfamethoxazole from wastewater.

## Introduction

The emergence of recently detected pollutants in potable water supplies continues to be a major concern for human health and the environment. Emerging pollutants (or contaminants of emerging concern [CECs]) are compounds that are detected at low levels in surface water and are not commonly monitored/regulated but may have an impact on aquatic life, ecosystems, and human health.^1,2^ The group of CECs consists mainly of antibiotics,^3^ pharmaceuticals,^4^ steroids,^5^ endocrine disruptors,^6^ hormones,^7^ industrial additives,^8^ chemicals from personal care products,^9^ and other distinct chemicals.^10^ Studies have also identified microbeads and microplastics as contributors to this category.^11,12^ Among the array of CECs, antibiotics and pharmaceuticals merit particular attention. Their presence in aquatic environments signals a potential for bioaccumulation within aquatic species. The subsequent consumption of these species by humans poses a considerable health concern, especially given the limited understanding of the potential health outcomes. At the same time, the presence of antibiotics in surface water can lead to the development of antibiotic-resistant bacteria. Unfortunately, commonly used adsorbents such as activated charcoal,^13^ zeolites,^14^ or clays^15^ have a low affinity or adsorption capacity toward these compounds, and, therefore, their removal by common wastewater treatment plants is insufficient.^16,17^ For these reasons, materials for capturing CECs from water are in high demand.

Metal–organic frameworks (MOFs) are porous coordination polymers consisting of metal nodes connected through organic ligands. Their chemical and topological variability can be utilized for gas storage and separation, catalysis, sensing, and medicinal applications. Due to their high specific surface area, their use for adsorption is likely the most prominent. The adsorption properties of MOFs can be enhanced by their modification with various functional groups.^18^ This is mostly achieved by a post-synthetic modification^19^ or the introduction of additional functional groups onto the organic ligands.^20^ The majority of the ligands used for the construction of MOFs contain carboxylic groups since they can coordinate the metal centers of all valences and form MOFs with predictable structures. Carboxylate-based MOFs also represent one of the most intensively studied groups of materials for the removal of CECs.^21−23^ However, many carboxylate-based MOFs are not stable in an aqueous environment,^24,25^ which limits their environmental applications.

Phosphinate groups bind to high-valent metals with a stronger bond than commonly used carboxylic groups. Since they also have only two accessible oxygen atoms for bonding, the formed structures are more predictable than in the case of phosphonate ligands with three accessible oxygen atoms.^26,27^ In recent years, we have presented a series of MOFs called ICR (Institute of Inorganic Chemistry Řež) derived from ligands containing phosphinate groups.^28,29^ Our research has demonstrated that, similar to carboxylates, phosphinate ligands can also be employed to form predictable structures. A phosphinate group possesses an additional alkyl or aryl group bonded to the phosphorus atom. In the case of the ICR-2 family of MOFs, this pendant alkyl or aryl group is facing the pore; therefore, this substituent can be used for tuning the chemical nature of the pore walls. Previously, we demonstrated that the pore size can be adjusted based on the length of the ligand and bulkiness of the substituent on the phosphorus atom while keeping the structure isoreticular.^30^

**Figure 1:**
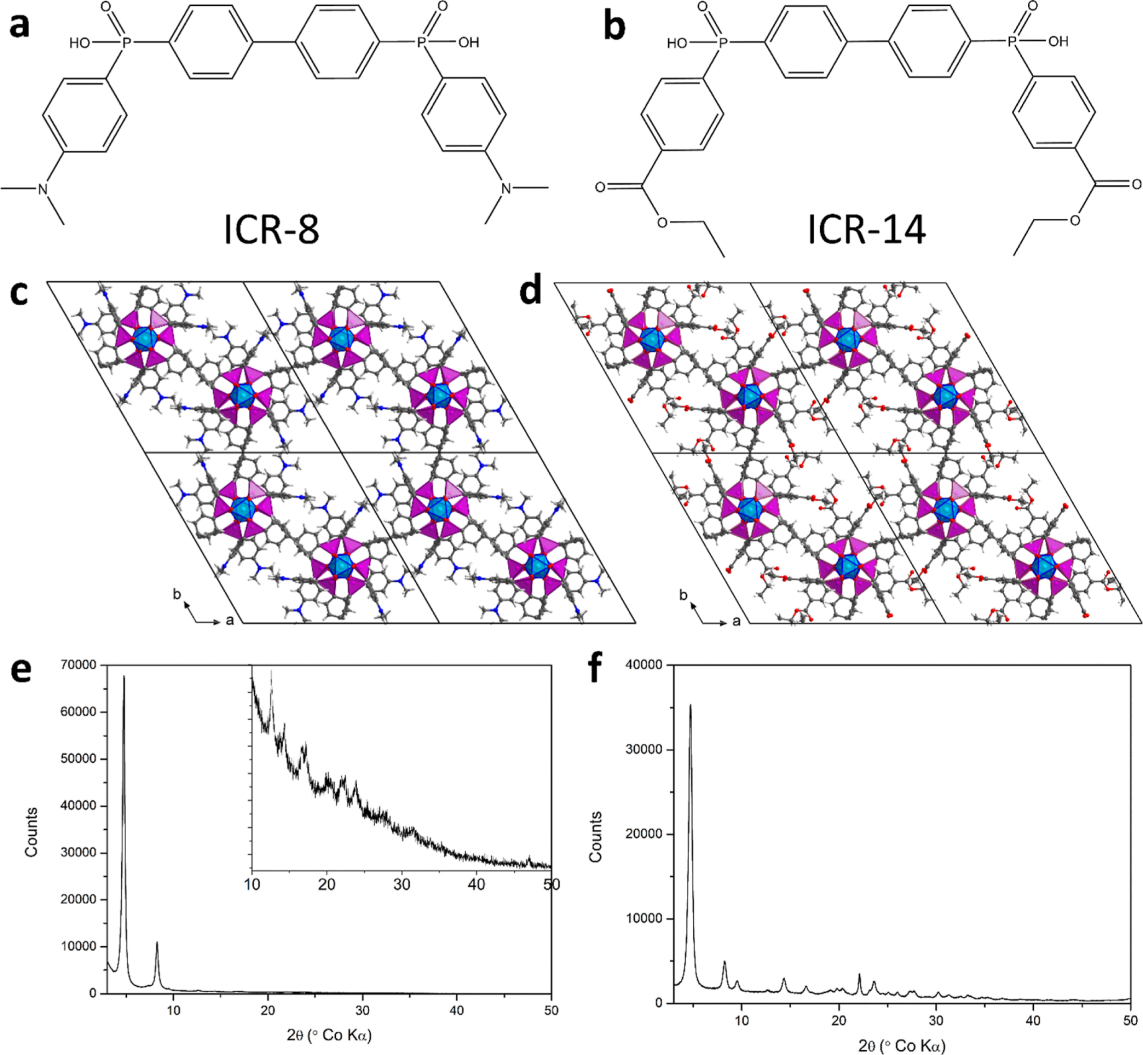
Phosphinate ligands forming the structures of the presented MOFs—H_2_BBP(Ph-NMe_2_) (a) and H_2_BBP(Ph-COOEt) (b); handmade models of honeycomb patterns of the 1D pores of ICR-8 (c) and ICR-14 (d) running along the *c*-axis; PXRD patterns of ICR-8 (e) and ICR-14 (f). Color coding is as follows: octahedrally coordinated iron atoms (light blue), phosphinate tetrahedra (magenta), O (red), C (gray), N (blue), and H (white).

The pollutant capture properties of the materials are determined not only by the specific surface area but also by the affinity of the pollutant to the surface.^31^ In contrast with conventional carboxylate-based MOFs, the ICR MOFs based on phosphinate linkers enable better tuning of the chemical nature of the pores by introducing additional non-coordinating functional groups on alkyl/aryl pendant groups on P atoms. In this work, we present the synthesis of two new phosphinate Fe-MOFs (ICR-8 and ICR-14) with structures isoreticular to ICR-7 (Figure 1) that contain −NMe_2_ and −COOEt groups oriented toward the pores. The primary objective is to demonstrate the influence of these functional groups on the adsorption of pollutants. Testing of pollutant removal was carried out on model pharmaceuticals, namely, diclofenac (DCF, a sodium salt-form analgesic and non-steroidal anti-inflammatory drug), cephalexin (CEP, a first-generation antibiotic), and sulfamethoxazole (SMX, an antibiotic), to determine how the functional groups affect the adsorption of the particular pollutants.

## Experimental Section

### Materials and Instrumental Methods

The description of used materials and instrumental methods can be found in the Supporting Information.

### Preparation of ICR-7

ICR-7 was prepared using a procedure reported earlier.^30^

### Preparation of ICR-8

A Teflon-lined stainless steel autoclave (Berghof DAB-2) was charged with 20.8 mg (0.040 mmol) of H_2_BBP(Ph-NMe_2_) and 5 mL of EtOH. The mixture was ultrasonicated for 10 min, after which 5.4 mg (0.020 mmol) of FeCl_3_·6H_2_O and an additional 5 mL of EtOH were added. The suspension was heated to 180 °C under autogenous pressure for 24 h. After cooling down to room temperature, the resulting powder was centrifuged (Hettich Rotina 380 R, 10 000 rpm, 10 min) and washed with ethanol, water, and acetone three times.

### Preparation of ICR-14

A Teflon-lined stainless steel autoclave (Berghof DAB-2) was charged with 41.8 mg (0.080 mmol) of H_2_BBP(Ph-COOH), 10.8 mg (0.040 mmol) of FeCl_3_·6H_2_O, and 5.0 mL of ethanol. The mixture was heated to 250 °C under the autogenous pressure for 24 h. After cooling down to room temperature, the resulting powder was centrifuged (Hettich Rotina 380R, 10 000 rpm, 10 min) and washed with ethanol, water, and acetone three times.

### Gas Adsorption Measurement

Adsorption isotherms of argon at 87 K were recorded using a 3P micro 300 instrument (3P Instruments) equipped with CryoTune units. Prior to the measurement, the sample was degassed at 100 °C for 24 h under dynamic vacuum. The specific surface area was calculated using the Brunauer–Emmett–Teller (BET) analysis of the 0.005–0.1 *p*/*p*_0_ range, and the distributions of pore sizes were calculated using the density functional theory (DFT) method, as provided by the 3P Instruments software.

### Adsorption of Pollutants

Before the experiments, the adsorbents were activated at 90 °C under a dynamic vacuum for 24 h, and the solutions of pharmaceuticals were tempered at 25 ± 1 °C. All experiments were done with solutions containing a single pollutant. The pH during all adsorption tests was natural without any adjustments. Activated carbon (DARCO in powdered form [100 mesh particles] from Sigma-Aldrich) was used as a comparative standard adsorbent material.

The adsorption kinetics were carried out with an initial pharmaceutical concentration of 100 mg L^–1^. 100 mL SIMAX reagent bottles were loaded with 5 mg of an adsorbent and sonicated in 12.5 mL of water to break particle aggregates. Then, 12.5 mL of a pollutant solution (200 mg L^–1^) was added. The reaction mixture was placed in a thermostatic box with a constant temperature of 25 ± 1 °C and shaken (190 rpm) using a horizontal shaker. At predefined times, 0.2 mL aliquots were taken and filtered using microfilters (Whatman PTFE membrane with 0.2 μm pores), and the pharmaceutical concentration was determined using high-performance liquid chromatography with diode array detection (HPLC-DAD) analysis (for specifications regarding the instrumentation and setup of the analysis, see the Supporting Information).

The adsorption isotherms were determined using an analogous procedure, with the difference being the initial concentrations of the pharmaceuticals ranging from 10 to 100 mg L^–1^. The samples were taken and analyzed after 24 h.

All adsorption experiments were conducted in duplicate to ensure reproducibility. Both kinetic data and adsorption isotherms were fitted using the pseudo-second-order kinetic model, Langmuir isotherm, Freundlich isotherm, and Sips isotherm (for detailed mathematical models and expressions, see the Supporting Information).

### Adsorbent Stability Testing

The adsorbents collected after the measurement of adsorption isotherms of pollutants were washed three times with acetone, air-dried, and re-activated. Subsequent analyses, including powder X-ray diffraction (PXRD) patterns and argon adsorption isotherms, were then conducted. The concentration of linkers released from the adsorbents was quantified using HPLC analysis or inductively coupled plasma mass spectrometry (ICP–MS) (see the Supporting Information for details).^32,33^ The linker release upon shaking in pure water was also assessed.

## Results and Discussion

### Synthesis and Characterization

**Scheme 1:**
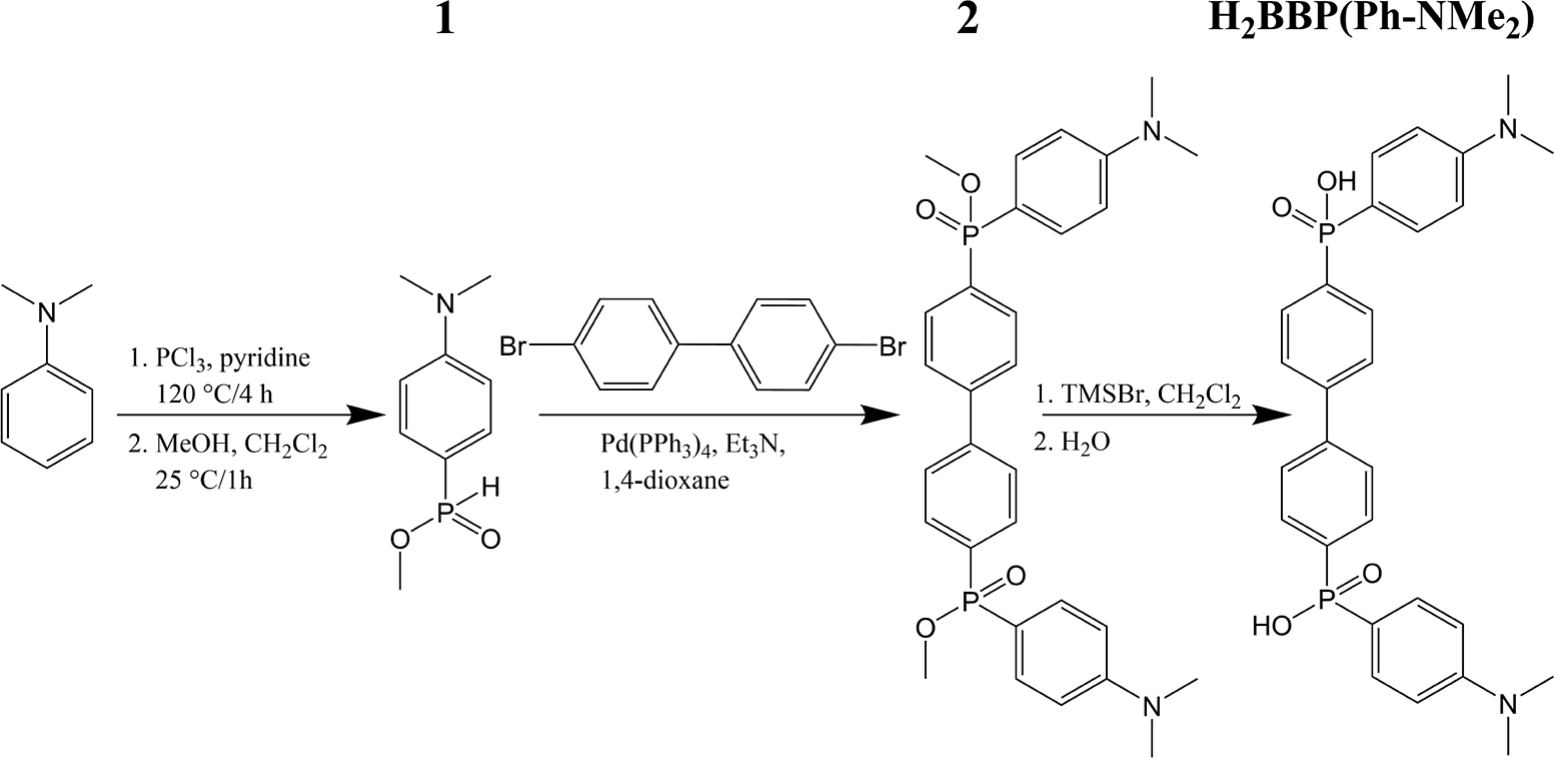
Synthesis of H_2_BBP(Ph-NMe_2_)

**Scheme 2:**
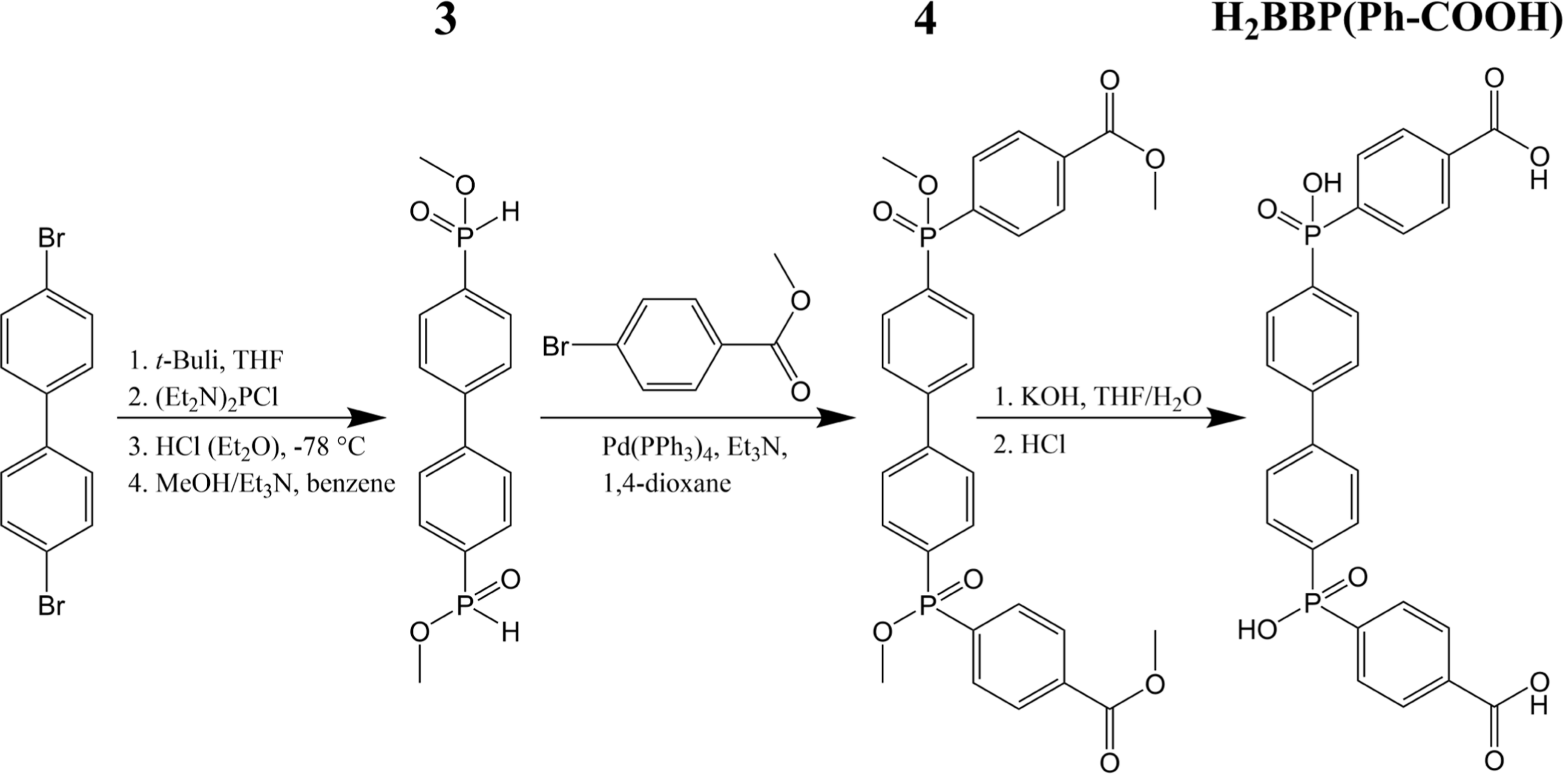
Synthesis of H_2_BBP(Ph-COOH)

The presented MOFs are based on functionalized H_2_BBP(Ph) linkers that have not been synthesized previously. The derivative with −NMe_2_ functional groups (**2**) was prepared by a palladium coupling reaction from 4,4′-dibromobiphenyl and methyl 4-(*N*,*N*-dimethylamino)phenylphosphinate (**1**) that was synthesized according to a published method using *N*,*N*-dimethylaniline (Scheme 1).^34^ The derivative with carboxylate groups (**4**) was synthesized through palladium coupling from dimethyl biphenyl-4,4′-diphosphinate (**3**) and methyl 4-bromobenzoate. The phosphinate group was attached to biphenyl by lithiation of 4,4′-dibromobiphenyl, followed by the reaction with bis(diethylamino)chlorophosphine and further transformation of the functional group to **1**, which is a valuable precursor for the attachment of a variety of aryl groups by a palladium coupling reaction (Scheme 2). The target linker molecules H_2_BBP(Ph-NMe_2_) and H_2_BBP(Ph-COOH) were prepared from the corresponding esters **2** and **4** by trimethylbromosilane- and KOH-assisted hydrolysis, respectively. MOFs ICR-8 and ICR-14 were prepared by solvothermal crystallization of the pertinent linker with FeCl_3_·6H_2_O in a PTFE-lined stainless steel autoclave. Both frameworks were formed in EtOH: ICR-8 at 180 °C and ICR-14 at 250 °C. The elemental composition of the materials (Table S2) measured using CHN combustion analysis and ICP–MS confirms the presence of the expected chemical composition. In the case of ICR-8, the greater difference between the expected and measured elemental content likely stems from the lower crystallinity of the sample, suggesting potential structural defects.

### Structure of ICR-8 and ICR-14

The PXRD patterns of both ICR-8 (Figure 1e) and ICR-14 (Figure 1f) possess a relatively broad peak profile. In the case of ICR-8, the intensity of diffraction peaks at high angles was extremely low; there was almost no observed diffraction peak below the resolution of *d* = 3 Å. Unfortunately, the attempts to prepare samples with improved crystallinity were not successful.

Both PXRD patterns can be fitted by a hexagonal unit cell derived from previously published data for ICR-7 (see Table 1 for comparison). The intensities of individual Bragg’s diffractions and also the refined unit cells suggest that the crystal structures of ICR-8 and ICR-14 follow the isoreticular MOFs ICR-6 and ICR-7,^30^ where a biphenyl spacer was also used as a middle part of the linker. Thus, it is quite reasonable to assume that the coordination backbone of both materials consists of 1D chains of octahedrally coordinated iron atoms forming eight-membered M-O-P-O-M-O-P-O rings (Figure S1), which is a prevalent coordination motif in phosphinate and phosphonate MOFs.^35^ These 1D coordination chains are interconnected by 4,4′-biphenylene groups (Figure S2), resulting in a 3D honeycomb arrangement (Figure 1c,d), similar to the structure of ICR-6 and ICR-7.

**Table 1:** Comparison of Unit Cells and Measured and Calculated Pore Characteristics of the Presented MOFs with ICR-7

compound name	ICR-7	ICR-8	ICR-14
linker	H_2_BBP(Ph)	H_2_BBP(Ph-NMe_2_)	H_2_BBP(Ph-COOEt)
a [Å]	25.0259(11)	24.774(6)	24.833(5)
c [Å]	9.5800(8)	9.420(11)	9.5555(10)
V [Å3]	5196.1(5)	5007(6)	5103.0(15)
specific surface area [m^2^ g^–1^]	927	677	411
external surface area [m^2^ g^–1^]^a^	494	391	261
pore diameter [nm]^b^	1.6	1.6	1.5
pore volume [cm^3^ g^–1^]	0.79	0.496	0.414
accessible surface area [m^2^ g^–1^]^c^	1097	422	37
pore-limiting diameter [nm]^c^	1.32	0.789	0.393
network-accessible geometric volume [cm^3^ g^–1^]^c^	0.64	0.409	0.347

a Calculated by *t*-plot.

b Most frequent pore diameter, for pore size distribution, see Figures S10–S12.

c Calculated by the PoreBlazer software assuming empty channels and considering van der Waals diameters of the atoms.

The presence of −NMe_2_ in ICR-8 is confirmed by the observation of a characteristic C–N vibration band at 1142 cm^–1^ in the Fourier transform infrared (FTIR) spectrum (Figure S5). The FTIR spectrum of ICR-14 (Figure S6) reveals intensive bands at 1716 and 1269 cm^–1^, indicating that the carboxylic groups are esterified by EtOH during the solvothermal crystallization of the MOF. The presence of ethyl ester groups on the linker molecules was further confirmed by the ^1^H NMR spectrum of ICR-14 dissolved in a deuterated solution of KOH (Figure S7). The esterification of carboxylic acids in the presence of Fe^3+^ ions is a well-known process,^36^ whereas phosphinic acids are typically resistant to direct esterification.^37^ This is the reason why only the carboxylic groups undergo esterification, and the coordination backbone in ICR-14 consists solely of coordinated phosphinates.

The structural study based on the measured PXRD patterns is practically impossible due to the large broadening of individual peaks, causing a high peak overlap. This situation is even more pronounced for ICR-8. Nevertheless, we sought to determine whether the assumption that the crystal structures of ICR-8 and ICR-14 are isoreticular to those of ICR-6 and ICR-7 is justified. The phenyl groups substituted with −NMe_2_ or −COOEt in the para position are larger than methyl or phenyl in ICR-6 and ICR-7, and similar to ICR-6 and ICR-7, these groups are anticipated to face the pores. However, the space inside the pore is limited, and it is necessary to determine whether the larger substituents can be arranged in a reasonable motif inside the pore. For that reason, structural models of both frameworks that were based on the crystal structure of ICR-7 were created. We added the dimethylamino and ethyl carboxylate groups, respectively, to the phenyl that faces the pore. In such manually created models, some of the atoms of the added functional groups overlapped, and this geometrical issue could not be resolved by a simple rotation of the ending groups. In the structures of ICR-6 and ICR-7, the 1D Fe columns are connected by biphenyl linkers that are parallel to each other. However, other configurations are possible; for instance, in the ICR-4 structure, the 1D Fe columns are connected through crossed biphenyl linkers.^30^ If the same structural motif is applied to the model of ICR-14, the ending ester groups facing the pore no longer overlap. Moreover, the free carboxylic ester groups provide several possibilities for creating an extended hydrogen-bonding system. This suggests that with minor modifications to accommodate the larger substituents, an isoreticular honeycomb configuration is achievable in both ICR-8 and ICR-14. A simple Rietveld fit using Jana2020 software with fixed atomic positions and atomic displacement parameters was performed. Considering that both models are just approximate and were created by manual placement and rotation of the linker in the unit cell, the theoretical PXRD patterns reasonably fit the measured data (Figures S3 and S4). The manually created structural models of ICR-8 (Figure 1c) and ICR-14 (Figure 1d) were also used for the calculation of the theoretical pore sizes using PoreBlazer software.^38^ The calculated values, together with the results of the analysis of adsorption isotherms of argon, are summarized in Table 1.

### Thermogravimetric Analysis

The thermogravimetric curves (Figures S8 and S9) confirmed the stability of ICR-8 and ICR-14 up to 350 °C. When compared to the previously published Fe-based phosphinate MOFs,^28,30^ they have lower thermal stability, which is likely due to the presence of additional functional groups. The decomposition of both MOFs is accompanied by the evolution of CO_2_ and H_2_O. In the case of ICR-8, the gradual decomposition begins at 300 °C. The evolution of H_3_PO_4_, which indicates the total breakup of the phosphinate backbone, was observed at 430–470 °C.

### Gas Adsorption Measurements

**Figure 2:**
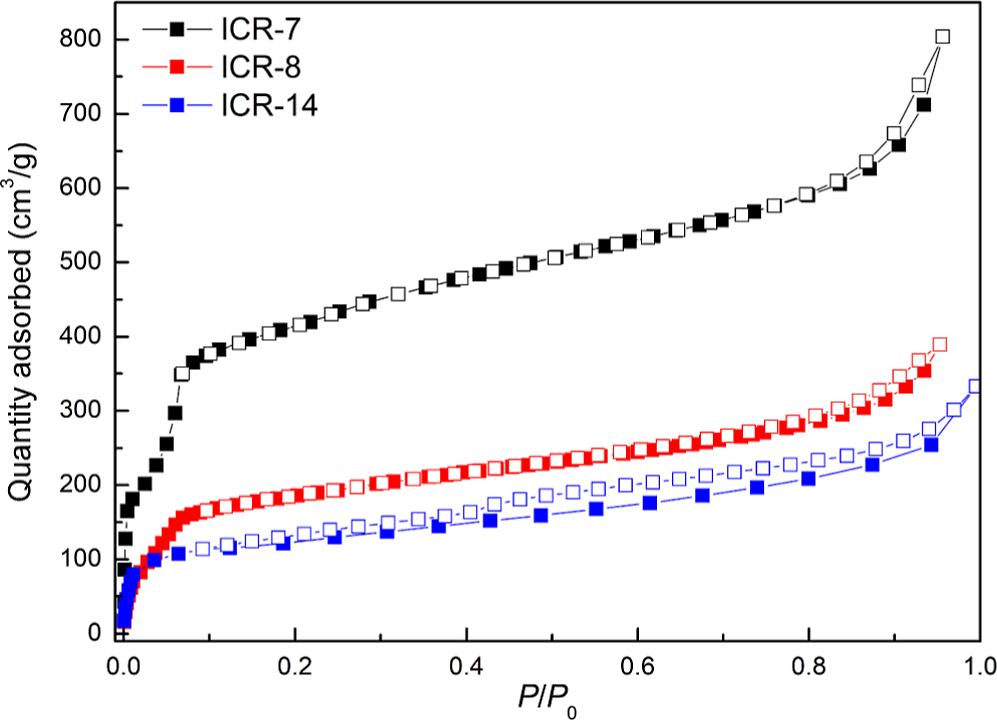
Adsorption isotherms of argon (87 K) for ICR-7, ICR-8, and ICR-14.

The porosity of the MOFs was probed by the adsorption of Ar. The adsorption isotherms (Figure 2) indicate the microporous nature of the MOFs with a considerable external surface area. The BET analysis yields a specific surface area (Table 1) of 677 and 411 m^2^ g^–1^ for ICR-8 and ICR-14, respectively. However, only 277 and 140 m^2^ g^–1^ for ICR-8 and ICR-14, respectively, are attributed to micropores; the remaining surface area is associated with the external surface of the MOFs. The micropore area of ICR-8 is below the theoretical values calculated using the PoreBlazer software, which suggests a significant blocking of micropores caused by the presence of functional groups or residual solvent molecules. In the case of ICR-14, however, the micropore surface area exceeds the calculated value, likely due to the presence of defects in the crystal structure.^39^ The presence of the functional groups leads to a lower specific surface area of the functionalized ICR MOFs compared to ICR-7. The pore size distributions (Figures S10–S12) calculated from the isotherms using the DFT method reveal predominant pore diameters of 16 and 15 Å for ICR-8 and ICR-14, respectively. These values are comparable to or lower than 16 Å for ICR-7 without functional groups in the pores. The larger size of pores in ICR-7 also results in the capillary condensation of the adsorbate, which is manifested by the typical Type IV shape of the adsorption isotherm in the 0–0.1 *p*/*p*_0_ range. However, it is necessary to note that the structural models of ICR-8 and ICR-14 are approximate, and discrepancies with measured data are to be expected. The measured pore size diameters of the studied MOFs are large enough to accommodate the selected pharmaceutical molecules, with diameters of 9.2, 11.3, and 10.1 Å for DCF, CEP, and SMX, respectively, as determined from the crystal structures of the particular compounds.^40−42^

### Adsorption of Pharmaceutical Pollutants

**Figure 3:**
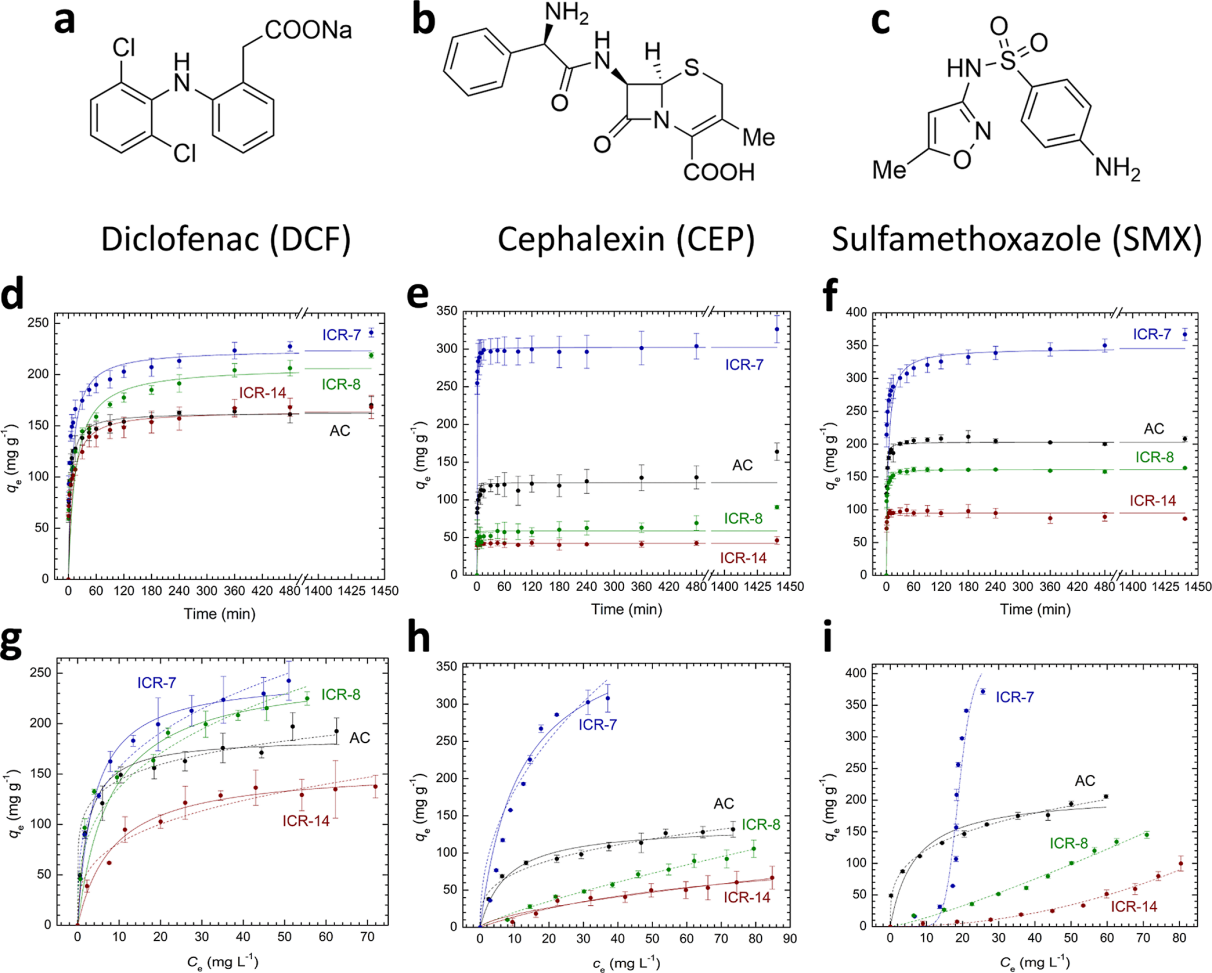
Structural formulas of the pharmaceutic pollutants used for the adsorption tests at natural pH values—DCF (a), CEP (b), and SMX (c); respective kinetics of adsorption on particular adsorbents (d–f) and adsorption isotherms (g–i) fitted by a Langmuir model (solid lines), Freundlich model (dashed line), or Sips model (dashed-dotted line, used in the case of the SMX/ICR-7 system).

The real-life applicability of ICR MOFs with functionalized pore walls was evaluated for the removal of pharmaceuticals that can be typically found in wastewater and are difficult to remove through established processes. The adsorption properties were studied on single-pollutant model solutions of DCF, CEP, and SMX. Their structures are depicted in Figure 3. To contextualize the performance of the newly prepared MOFs against other pollutants, we used commercial activated carbon (DARCO in powdered form [100 mesh particles] from Sigma-Aldrich, abbreviated as AC) for comparison.

The adsorption kinetic curves (Figure 3d–f) represent the mathematical fit of the pseudo-second-order kinetic model^43^ (see eq 2 in the Supporting Information). The parameters resulting from the fitting are summarized in Table 2.

**Table 2:** Adsorption Kinetic Parameters

pollutant	adsorbent	*Q*_max_ (mg g^–^^1^)	*k*_2_ × 10^3^ (g mg^–^^1^min^–^^1^)	*R*^2^
DCF	AC	162.9 ± 1.7	1.40 ± 0.21	0.999
	ICR-7	224.6 ± 3.9	0.54 ± 0.11	0.999
	ICR-14	164.5 ± 2.3	0.74 ± 0.11	0.999
	ICR-8	208.1 ± 3.9	0.31 ± 0.05	0.996
CEP	AC	122.9 ± 3.5	22.93 ± 7.77	0.856
	ICR-7	302.4 ± 2.3	13.11 ± 8.75	0.999
	ICR-14	42.2 ± 0.4	n.a^a^	n.a^a^
	ICR-8	58.8 ± 2.9	n.a^a^	n.a^a^
SMX	AC	202.9 ± 2.0	11.51 ± 1.27	0.981
	ICR-7	346.3 ± 4.1	0.69 ± 0.15	0.999
	ICR-14	94.9 ± 1.1	66.48 ± 13.66	0.970
	ICR-8	161.4 ± 0.5	8.06 ± 0.93	0.999

a Saturation was reached too fast to calculate the rate constant.

In the case of DCF (Figure 3d), the adsorption capacity *Q*_max_ decreases in the order ICR-7 (224.6 mg g^–1^) > ICR-8 (208.1 mg g^–1^) > ICR-14 (164.5 mg g^–1^) > AC (162.9 mg g^–1^). The presented MOFs have a higher adsorption capacity for DCF than AC; however, the lower rate constants (*k*_2_) suggest that the adsorption rate for these MOFs is slower than for AC, differing by nearly an order of magnitude. Nevertheless, in all instances, the adsorption is rapid enough to accommodate the majority of the pollutant within the first few minutes.

The adsorption kinetic curves (Figure 3e) and parameters for CEP show that saturation is reached in the first few minutes for the all studied adsorbents. The adsorption rate for ICR-7 is comparable to AC, while the adsorption processes on ICR-14 and ICR-8 are so rapid that the kinetic rate constant *k*_2_ cannot be determined using the pseudo-second-order kinetic model due to the absence of data points between the onset and the first sampling at 0.5 min. The highest adsorption capacity for CEP was reached with ICR-7 (302.4 mg g^–1^), compared to only 42.2 and 58.8 mg g^–1^ for ICR-14 and ICR-8, respectively.

In the case of SMX, more significant differences in adsorption kinetics (Figure 3f) were observed. The highest adsorption capacity was reached by ICR-7 (346.3 mg g^–1^). However, the pseudo-second-order rate constant indicates significantly slower adsorption compared to other adsorbents. ICR-8 has a slightly lower adsorption capacity for SMX (161.4 mg g^–1^) than AC (202.9 mg g^–1^); nonetheless, the rate of adsorption is comparable. ICR-14 has an adsorption capacity for SMX of only 94.9 mg g^–1^. Notably, saturation occurred almost immediately after the immersion of the adsorbent into the SMX solution.

In summary, ICR-7 reveals the highest uptake of all the studied pharmaceutical pollutants (Figure S13), but the adsorption proceeds slower than with AC. On the other hand, ICR-14 reveals the highest adsorption rates; however, its capacity to capture pollutants is limited compared to AC. While ICR-8 can adsorb a greater amount of DCF than AC, it falls short in absorbing other pollutants. Clearly, the adsorption characteristics do not depend only on the nature of the adsorbent but also on the specific pollutant.

The analyses of *Q*_max_ based on adsorption isotherms (Figure 3g–i) are in line with the results obtained from the analysis of the adsorption kinetics. In the case of DCF, the adsorption isotherms (Figure 3g) exhibit a Type I (or L-class) isotherm, where ICR-7 and ICR-8 display approximately 1.3 times greater adsorption capacities than AC, while ICR-14 has a similar capacity to AC. The Langmuir constants (*K*_L_) quantifying the affinity of the pollutant to the adsorbents (Table 3) decrease in the order AC > ICR-7 > ICR-8 > ICR-14, indicating that ICR-7 and ICR-8 possess higher adsorption capacities for DCF than AC despite the lower affinity. The affinity of DCF to the adsorbent mainly affects the adsorption rates, which are in line with the trends set by the *K*_L_ values. Interestingly, ICR-7 and ICR-8 adsorbents exhibit higher adsorption capacities for DCF than the commonly tested carboxylate-based MOF UiO-66 (*Q*_max_ = 189 mg g^–1^),^44^ while the performance of ICR-14 is roughly equivalent to that of UiO-66. Moreover, ICR-7 and ICR-8 exhibit higher adsorption capacities for DCF than some carbon-based adsorbents.^45−47^

**Table 3:** Parameters of the Adsorption Isotherms

		Langmuir model			Freundlich model		
pollutant	adsorbent	*Q*_max_ (mg g^–1^)	*K*_L_ (L mg^–1^)	*R*^2^	*K*_F_ [(mg g^–1^) (mg L^–1^)^−*n*^]	*n*	*R*^2^
DCF	AC	186.1 ± 6.1	0.452 ± 0.094	0.966	102.17 ± 0.81	6.73 ± 0.10	0.853
	ICR-7	246.9 ± 6.6	0.255 ± 0.032	0.986	80.51 ± 6.41	3.47 ± 0.29	0.975
	ICR-14	155.8 ± 5.5	0.116 ± 0.017	0.981	40.45 ± 6.02	3.29 ± 0.44	0.947
	ICR-8	253.8 ± 6.7	0.127 ± 0.019	0.998	64.94 ± 0.71	3.10 ± 0.03	0.947
CEP	AC	137.3 ± 5.6	0.128 ± 0.023	0.971	40.317 ± 0.45	3.62 ± 0.04	0.930
	ICR-7	411.6 ± 26.7	0.088 ± 0.019	0.995	64.85 ± 0.62	2.21 ± 0.02	0.976
	ICR-14	85.1 ± 21.7	0.013 ± 0.004	0.961	3.984 ± 1.12	1.58 ± 0.18	0.990
	ICR-8	n.a.	n.a.	0.992	3.23 ± 0.52	1.26 ± 0.06	0.991
SMX	AC	210.2 ± 15.4	0.149 ± 0.047	0.924	63.22 ± 2.59	3.54 ± 0.1	0.995
	ICR-7	424.1 ± 60.4^a^	n.a.	0.895	n.a.	n.a.	n.a.
	ICR-14	n.a.	n.a.	n.a.	0.01 ± 0.001	0.49 ± 0.003	0.997
	ICR-8	n.a.	n.a.	n.a.	0.90 ± 0.25	0.83 ± 0.05	0.998

a Based on the Sips mathematical model.^60^

As for the adsorption of CEP, the adsorption isotherms (Figure 3h) for AC and ICR-7 follow the Langmuir model, providing adsorption capacities of 137.3 and 411.6 mg g^–1^, respectively, which correspond to the values obtained from the analysis of adsorption kinetics. ICR-7 has a greater adsorption capacity than AC in spite of its lower *K*_L_ value. This indicates a higher affinity of CEP for AC than for ICR-7, elucidating the slower adsorption on ICR-7. The adsorption of CEP on the functionalized ICR MOFs follows the linear Henry’s adsorption model (C-type isotherm) typical for situations of low coverage of the adsorbent surface.^48,49^ The rapid adsorption of CEP on ICR-8 and ICR-14, surpassing that on ICR-7 or AC, hints at its enhanced affinity for the functionalized ICR MOFs; however, the linear shape of the respective isotherms suggests a surface coverage constraint due to the limited number of adsorption sites. The performance of ICR-7 in CEP removal is comparable to that of the previously studied PCN-777, which also followed the Langmuir model and revealed an adsorption capacity of 443 mg g^–1^.^50^ Other tested adsorbents, for example, carbon-based^51^ or inorganic^52,53^ materials, exhibited adsorption capacities below 100 mg g^–1^.

In the case of SMX adsorption, the studied ICR MOFs behave differently from AC. The adsorption isotherm (Figure 3i) for ICR-7 reveals a sigmoidal shape (Type V or S-type isotherm), which is typically associated with a limited affinity of the adsorbate to the adsorbent surface at very low adsorbate concentrations.^54,55^ After the surface is partially covered and modified by the adsorbed SMX molecules, the adsorption properties significantly improve. For example, this atypical behavior was observed during the adsorption of organic pollutants on zeolites.^56^ The adsorption capacity calculated from the adsorbed amount in the plateau region using the Sips mathematical model is 424.1 mg g^–1^. The behavior of ICR-8 is similar to its adsorption of CEP, indicating a limited number of adsorption sites on the surface, whereas the isotherm for ICR-14 has a concave shape (Type III isotherm), suggesting a weak interaction between SMX and ICR-14 and an exponential increase of the uptake after the adsorbent surface is partially covered by SMX molecules.^54,57^

In general, the diversity of adsorption isotherms implies that the adsorption of organic pollutants on MOFs involves intricate mechanisms acting across multiple active sites, such as ionizable functional groups and hydrophobic moieties.^58,59^ For this reason, the adsorption capacities of the materials are not directly proportional to their specific surface area. Significant influence is also related to the size and shape of the pores and the strength of interactions between the adsorbent and adsorbate.

### Stability of MOFs upon the Adsorption of Pollutants

**Figure 4:**
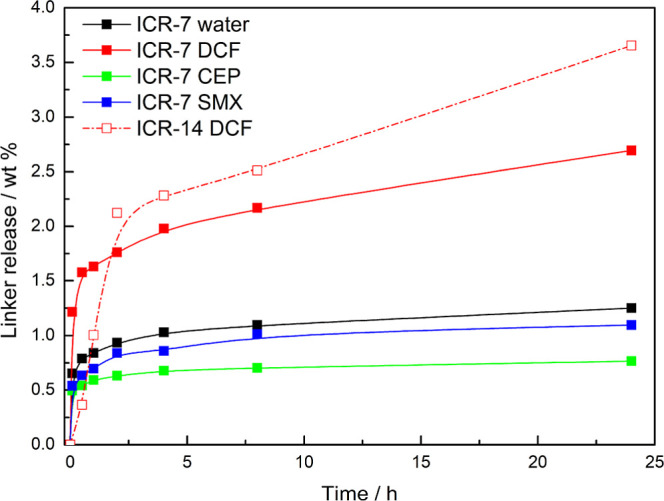
Linker release from ICR MOFs in the solutions of pharmaceutical pollutants and pure water (blank). Only data for ICR-7 (all solutions) and ICR-14 (DCF solution) are included because in other cases, the linker concentration was below the detection limit.

The PXRD patterns of the MOFs recorded after the adsorption and desorption of pollutants (Figures S14–S16) indicate that they all retain their original structures without the formation of any other crystalline phase or loss of crystallinity. However, previous studies carried out on Zr-MOFs suggest that even a significant linker loss does not have to lead to the collapse of the framework. The linker molecules were detected by HPLC in the solutions of pollutants after the adsorption experiments to quantify the linker release. In the case of ICR-14, the ICP–MS analysis was performed instead of HPLC to detect the linker in all potential forms, given the possibility of partial de-esterification of H_2_BBP(Ph-COOEt). Interestingly, partial release of the linker was detected for ICR-7 in all tested solutions (Figure 4). The highest concentration was recorded for DCF solution with a pH of 7.0 ± 0.1, which corresponds to a 2.7% loss of the linker content based on the theoretical formula. The reason for lower concentrations of the linker in SMX and CEP solutions might be attributed to a lower pH of 5.0 ± 0.3; this can be corroborated by similar concentrations of the released linker in pure water with a similar pH. These observations suggest that the linker release is affected mainly by pH.

In the case of ICR-14, the released linker was detected only in the solution of DCF (0.5 mg L^–1^ of P in the solution, which corresponds to 3.7% of the total amount of the linker). In the other solutions or pure water, no dissolved linker was found, which indicates that ICR-14 is stable at a pH of 5.0. However, at a pH of 7.0, partial decomposition is already occurring. In contrast, for ICR-8, we observed no linker release, even in the DCF solution at a pH of 7.0.

In summary, the hydrolytic stability of the tested ICR MOFs increases in the order ICR-7 < ICR-14 < ICR-8. Notably, all of them reveal better hydrolytic stability than carboxylate-based UiO-66, which loses about 10% of the linker after 4 h at a pH of 7.0.^32^ In the case of carboxylate-based Zr-MOFs, hydrolytic stability is typically increased by introducing hydrophobic ligands.^61,62^ However, in this study, a similar effect is achieved by introducing polar functional groups into the structure. The increased hydrolytic stability of the functionalized ICR MOFs might result from the enhanced protection of metal centers within the frameworks.

The argon adsorption isotherms were recorded again after the adsorption and consecutive desorption of the pollutants to gain a comprehensive understanding of the changes caused by the adsorption of pharmaceutical pollutants (Figures S17–S19). The desorption was carried out by washing the solids with 10 mL of acetone three times, and its completion was confirmed by HPLC analysis of the solids dissolved in 0.1M NaOH, where no traces of residual pollutants were detected. The adsorption isotherms of the ICR MOFs after their use for the removal of pharmaceuticals confirm that the materials retain their porous nature. However, their specific surface area (Table S3) decreases to 344–512 m^2^ g^–1^. ICR-14 treated with the DCF solution reveals a higher volume of adsorbed gas than before the treatment (Figure S19). This phenomenon can be attributed to its relatively significant linker loss (about 3.7%), the most substantial among all tested ICR MOFs, leading to the formation of structural defects known to significantly affect gas adsorption.^39^

## Conclusions

We synthesized novel phosphinate linkers, leading to the creation of MOFs ICR-8 and ICR-14, with structures stemming from the isoreticular ICR-7 described previously. The presented MOFs contain additional −NMe_2_ and −COOEt functional groups pointing inside the pores, which significantly change the chemical nature of the pore walls. During the synthesis of ICR-14, the −COOH group of the linker is esterified by the solvent (ethanol). When tested for the removal of three model emerging pharmaceutical pollutants (DCF, CEP, and SMX), the functionalized MOFs displayed enhanced affinity, resulting in rapid adsorption rates. However, the adsorption capacity diminished, possibly due to the lower specific surface area and competitive interactions of the surface with water molecules. This highlights the complex processes involved in the adsorption of pollutants on MOFs and provides valuable insights into the rational design of adsorbents for pollutants of emerging concern. In spite of the lower adsorption capacity of the functionalized MOFs, they have greater hydrolytic stability than ICR-7. All the three tested MOFs retained their original structure and porous nature after the adsorption and desorption of pollutants, and compared to UiO-66, only a negligible amount of linker was released from the materials. This makes phosphinate MOFs suitable for this type of applications.

A preprint version of this work was submitted to the ChemRxiv preprint server prior to the submission.^63^

## Supporting Information Available

Synthesis and characterization of precursors, descriptions of the instrumental methods, mathematical models employed for experimental data, elemental analysis, Rietveld fitting of PXRD patterns, FTIR spectra, thermogravimetric analysis (TGA) curves, pore size distributions, and stability studies (PDF)
