# Supplementary material for: Linker-Functionalized Phosphinate Metal–Organic Frameworks: Adsorbents for the Removal of Emerging Pollutants
Source: Inorg Chem. 2023 Sep 8;62(38):15479–89. doi: 10.1021/acs.inorgchem.3c01810 (PMC10523435; doi:10.1021/acs.inorgchem.3c01810)
Supplement: Supplementary file 1 — ic3c01810_si_003.pdf [file ic3c01810_si_003.pdf]

Linker functionalized phosphinate metal-organic frameworks: Adsorbents for the removal of emerging pollutants – Supporting information

*Soňa Ondrušová,[†,‡] Daniel Bůžek,[†,$] Matouš Kloda,[†] Jan Rohlíček,[§] Slavomír Adamec,[$] Miroslav Pospíšil,[ǁ] Pavel Janoš,[$] Jan Demel,[†] Jan Hynek,[†]**

[†]Institute of Inorganic Chemistry of the Czech Academy of Sciences, Husinec-Řež 1001, 250 68 Řež, Czech Republic; E-mail: hynek@iic.cas.cz

[‡]Department of Inorganic Chemistry, Faculty of Science, Charles University, Hlavova 2030, 128 40 Prague, Czech Republic

[$]Department of Environmental Chemistry and Technology, Faculty of Environment, Jan Evangelista Purkyně University in Ústí nad Labem, Pasteurova 3632/15, 400 96 Ústí nad Labem, Czech Republic

[§]Institute of Physics of the Czech Academy of Sciences, Na Slovance 1999/2, 182 21 Prague, Czech Republic

[ǁ]Department of Chemical Physics and Optics, Faculty of Mathematics and Physics, Charles University, Ke Karlovu 3, 121 16 Prague, Czech Republic

Table of contents

Materials 1
Synthesis of the ligands 2
Instrumental methods 5
Mathematical models for adsorption experiments 6
Supplementary Figures and Data 7
Elemental analysis 7
Infrared spectra 10
Thermogravimetric analysis 13
Porosity determination 15
Stability studies 17
Chemical analytical data for MOF precursors 22

Materials

4,4ˈ-dibromobiphenyl (TCI Chemicals), *t*-BuLi (1.9M solution in pentane), hydrogen chloride (2N solution in diethyl ether), methyl 4-bromobenzoate, phosphorus trichloride (all Acros

Organics), $Pd(PPh_3)_4$ (abcr), *N,N*-dimethylaniline, pyridine, benzene (anhydrous), trimethylsilyl bromide, $FeCl_3.6H_2O$, (all Sigma-Aldrich), bis(*N,N*-diethylamino)chlorophosphine (Alfa Aesar), ethanol (absolute, Fischer Chemical), sodium sulfate, sodium hydroxide, diethyl ether, acetone (all Lach:Ner, Czech Republic) were used as purchased. 1,4-dioxane (water-free, VWR Chemicals), methanol (anhydrous, VWR Chemicals) and dichloromethane (HPLC grade, Fisher Scientific) for syntheses were dried using solvent purifier SP-1 (LC Technology Solutions). Triethylamine (Sigma-Aldrich) was freshly distilled with Na under Ar. Tetrahydrofuran (Lach:Ner, Czech Republic) was dried by refluxing with sodium and benzophenone until blue, and then distilled. The synthesis of phosphinate esters was performed under Ar using standard Schlenk technique. Column chromatography was performed on Sigma-Aldrich 60 (70-230 mesh, 60 Å) silica gel. Sulfamethoxazole (SMX, antibiotic, Pharmaceutical secondary standard), cephalexin (CEP, first-generation cephalosporin antibiotic, nonspecific purity), and diclofenac sodium salt (DCF, analgesics and anti-inflammatory drug, Pharmaceutical secondary standard), all from Sigma-Aldrich, were used as model pharmaceutics for testing of pollutant removal. Mobile phases for HPLC were made of acetonitrile (HiPerSolv Chromanorm, Super gradient for UPLC/UHPLC, VWR International) and phosphate buffer solution (pH 2.5) made by dissolving sodium dihydrogen phosphate dihydrate (p. a.) and ortho-phosphoric acid (85%), both Lach:Ner, Czech Republic, in ultrapure water from Direct-Q 3 UV water purification system.

Synthesis of the ligands

Preparation of methyl 4-(*N,N*-dimethylamino)phenylphosphinate (3). A three-necked flask was three times evacuated and flushed with Ar and charged with 5 mL of freshly distilled dimethylaniline (39 mmol). After cooling with an ice/potassium oxalate bath, 10 mL of phosphorus trichloride (114 mmol) and 9 mL of pyridine (112 mmol) were added. The reaction mixture was heated at 120 °C for 4 h. The excess of phosphorus trichloride was distilled off and the residue was dissolved in 10 mL of CH_2Cl_2. The solution was cooled by an ice bath and 22 mL of MeOH (0.54 mol) was slowly added. The reaction mixture was stirred for 1 h at RT. The solvent was removed by rotary evaporation and the solid residue was dissolved in CH_2Cl_2 and washed with water 3 times. The organic fraction was dried over anhydrous $MgSO_4$, filtered and evaporated to dryness. The crude product was purified by column chromatography on SiO_2 using 95:5 dichloromethane/methanol mixture as an eluent.

Yield: 3.57 g (46 %).

^{1}H NMR ($CDCl_3$): δ 7.58 (dd, $^3J_{PH}$ = 13.0, $^3J_{HH}$ = 9.0 Hz, 2H), 7.48 (d, $^1J_{PH}$ = 564 Hz, 1H), 6.71 (dd, $^3J_{HH}$ = 9.0 Hz, $^4J_{PH}$ = 2.8, 2H), 3.72 (d, $^3J_{PH}$ = 12.3, 3H), 3.02 (s, 6H).

^{31}P{^{1}H} NMR ($CDCl_3$): δ 29.5.

Preparation of dimethyl biphenyl-4,4'-bis(4-(*N,N*-dimethylamino)phenylphosphinate) (4). A Schlenk tube was charged with 1.86 g of 4,4'-dibromobiphenyl (6.0 mmol), 3.57 g of

methyl 4-(N,N-dimethylamino)phenylphosphinate (18 mmol) and 1.1 g of $Pd(PPh_3)_4$ (0.95 mmol), three times evacuated and flushed with Ar. Then 60 mL of dry 1,4-dioxane was added followed by addition of 2.0 mL of dry triethylamine (14 mmol). The reaction mixture was stirred at 60 °C for 96 hours. After cooling to room temperature, the formed precipitate was removed by filtration and the filtrate was evaporated to dryness. The solid residue was dissolved in dichloromethane and washed with water 3 times. The organic fraction was dried over anhydrous $MgSO_4$, filtered and evaporated to dryness. The product was isolated by column chromatography on SiO_2 using 95:5 dichloromethane/methanol mixture as an eluent.

Yield: 2.92 g (89 %).

^{1}H NMR ($CDCl_3$): δ 7.84 (dd, $^3J_{PH}$ = 11.9, $^3J_{HH}$ = 8.2 Hz, 4H), 7.66 (dd, $^3J_{PH}$ = 11.6, $^3J_{HH}$ = 8.8 Hz, 4H), 7.61 (dd, $^3J_{HH}$ = 8.2 Hz, $^4J_{PH}$ = 2.8, 4H), 6.71 (dd, $^3J_{HH}$ = 8.8 Hz, $^4J_{PH}$ = 2.8, 4H), 3.74 (d, $^3J_{PH}$ = 11.1, 6H), 3.01 (s, 12H).

^{31}P{^{1}H} NMR ($CDCl_3$): δ 35.3.

Preparation of biphenyl-4,4ˈ-bis(4-(*N,N*-dimethylamino)phenylphosphinic acid) (H_2BBP(Ph-NMe_2). A round-bottom flask was charged with 2.00 g of dimethyl biphenyl-4,4ˈ-bis(4-(N,N-dimethylamino)phenylphosphinate) (3.6 mmol), evacuated and flushed with argon three times, and then 200 mL of CH_2Cl_2 was added followed by a dropwise addition of 1.1 mL of trimethylsilyl bromide (8.3 mmol). The reaction mixture was stirred at 40 °C for 16 h. After that, the solvent was removed by rotary evaporation. The solid residue was suspended in water, filtered off and washed with a small amount of acetone and diethyl ether.

Yield: 1.18 g (62 %).

^{1}H NMR (DMSO-d6): δ 7.79–7.62 (m, 8H), 7.49 (dd, $^3J_{PH}$ = 11.3, $^3J_{HH}$ = 8.6 Hz, 4H), 6.70 (dd, $^3J_{HH}$ = 8.8 Hz, $^4J_{PH}$ = 2.5, 4H), 2.90 (s, 12H).

^{31}P{^{1}H} NMR ($CDCl_3$): δ 25.2.

Preparation of dimethyl biphenyl-4,4ˈ-diphosphinate (1). A two-necked round-bottom flask was charged with 3.31 g of dibromobiphenyl (10.6 mmol), three times evacuated and flushed with Ar, and dissolved in 80 mL of THF. The solution was cooled by a CO_2(s)/EtOH cooling bath and 22.5 mL of 1.9M solution of *t*-BuLi (42.8 mmol) in pentane was slowly added. The solution was stirred for 30 min upon cooling by CO_2(s)/EtOH bath and then it was replaced for an ice bath and stirred for additional 90 min. After that, a solution of 8.95 g of bis(diethylamino)chlorophosphine (42.5 mmol) in 20 mL of THF was added. After 1 h of stirring upon cooling, the cooling bath was removed and the reaction mixture was stirred for additional 16 h at RT. The reaction mixture was cooled by a CO_2(s)/EtOH cooling bath and 85 mL of 1M solution of HCl (85 mmol) in diethyl ether was added. The solution was stirred 1 h upon) cooling and then 20 h at RT. The formed slurry was separated by canula filtration and the filtrate was evaporated *in vacuo*. The evaporated solid was dissolved in benzene. In a

Schlenk tube, 8.4 mL of MeOH (208 mmol) was mixed with 11.9 mL of dry triethylamine (85 mmol) under Ar atmosphere. The mixture was cooled in an ice bath and the benzene solution from the flask was added slowly. The formed precipitate was filtered off and the filtrate was evaporated. The oily product was mixed with water and stirred for 1 h at RT. After that, the product was extracted by dichloromethane, dried over Na_2SO_4, filtered and rotary evaporated.

Yield: 2.40 g (73 %).

^{1}H NMR ($CDCl_3$): δ 7.89 (dd, $^3J_{PH}$ = 13.4 Hz, $^3J_{HH}$ = 8.2 Hz, 4H); 7.76 (dd, $^3J_{HH}$ = 8.2 Hz, $^4J_{PH}$ = 3.1 Hz, 4H); 7.62 (d, $^1J_{PH}$= 570 Hz, 2H); 3.83 (d, $^3J_{PH}$= 12.0 Hz, 6H).

^{31}P{^{1}H} NMR ($CDCl_3$): δ 26.9.

Preparation of dimethyl biphenyl-4,4'-bis(4-methoxycabonylphenylphosphinate) (2). A Schlenk tube was charged with 5.00 g of methyl 4-bromobenzoate (23 mmol) and 2.69 g of $Pd(PPh_3)_4$ (2.3 mmol), three times evacuated and flushed with Ar. Then 12 mL of dry 1,4-dioxane was added followed by addition of 3.9 mL of dry triethylamine (28 mmol) and 2.4 g of dimethyl biphenyl-4,4'-diphosphinate (7.7 mmol) dissolved in 20 mL of dry dioxane. The reaction mixture was stirred at 60 °C for 96 hours. After cooling to room temperature, the formed precipitate was removed by filtration and the filtrate was evaporated to dryness. The product was separated by column chromatography on SiO_2 using 95:5 dichloromethane/methanol mixture as an eluent and purified by dissolving in dichloromethane and precipitation with diethyl ether.

Yield: 1.77 g (40 %).

^{1}H NMR ($CDCl_3$): δ 8.11 (dd, $^3J_{HH}$ = 8.2 Hz, $^4J_{PH}$ = 1.9 Hz, 4H); 7.92–7.87 (m, 8H); 7.66 (dd, $^3J_{HH}$ = 8.2 Hz, $^4J_{PH}$ = 1.9 Hz, 4H); 3.92 (s, 6H); 3.80 (d, $^2J_{PH}$ = 12.0 Hz, 6H).

^{31}P{^{1}H} NMR ($CDCl_3$): δ 32.2.

^{13}C{^{1}H} NMR ($CDCl_3$): δ 166.3, 144.0 (d, $^4J_{PC}$ = 3 Hz), 135.9 (d, $^1J_{PC}$ = 135 Hz), 133.6 (d, $^4J_{PC}$ = 3 Hz), 132.5 (d, $^3J_{PC}$ = 10 Hz), 131.8 (d, $^3J_{PC}$ = 10 Hz), 130.7, 129.7 (d, $^2J_{PC}$ = 13 Hz), 127.7 (d, $^2J_{PC}$ = 13 Hz), 52.6, 51.9 (d, $^2J_{PC}$ = 6 Hz).

Preparation of biphenyl-4,4'-bis(4-carboxyphenylphosphinic acid) (H_2BBP(Ph-COOH) A round-bottom flask was charged with 2.00 g of dimethyl biphenyl-4,4'-bis(4-(N,N-dimethylamino)phenylphosphinate) (3.6 mmol), evacuated and flushed with argon three times, and then 200 mL of CH_2Cl_2 was added followed by a dropwise addition of 1.1 mL of trimethylsilyl bromide (8.3 mmol). The reaction mixture was stirred at 40 °C for 16 h. After that, the solvent was removed by rotary evaporation. The solid residue was suspended in water, filtered off and washed with a small amount of acetone and diethyl ether.

Yield: 1.37 g (94 %).

^{1}H NMR (DMSO-d6): δ 7.98 (dd, $^3J_{HH}$ = 8.2, $^4J_{PH}$ = 2.6 Hz, 4H), 7.90–7.67 (m, 12H).

$^{31}P\{^{1}H\}$ NMR (DMSO-d6): δ 22.6.

$^{13}C\{^{1}H\}$ NMR (DMSO-d6): δ 167.3, 142.7 (d, $^{4}J_{PC}$ = 3 Hz), 140.0 (d, $^{1}J_{PC}$ = 132 Hz), 134.5 (d, $^{1}J_{PC}$ = 136 Hz), 133.9 (d, $^{4}J_{PC}$ = 3 Hz), 132.3 (d, $^{3}J_{PC}$ = 10 Hz), 131.7 (d, $^{3}J_{PC}$ = 10 Hz), 129.8 (d, $^{2}J_{PC}$ = 13 Hz), 127.7 (d, $^{2}J_{PC}$ = 13 Hz).

Instrumental methods

^{1}H, ^{31}P and ^{13}C NMR spectra were measured on a JEOL 600 MHz NMR spectrometer. The chemical shifts were referenced to the residual ^{1}H and ^{13}C signal of the deuterated solvents. Powder X-ray diffraction (XRD) was measured using a PANalytical X'Pert PRO diffractometer in the reflexion setup equipped with a conventional Co X-ray tube (40 kV, 30 mA). Qualitative analysis was performed with the HighScorePlus software package (PANalytical, Almelo, The Netherlands, version 3.0). Thermal analyses (DTA/TGA) were carried out on a Setaram SETSYS Evolution-16-MS (Setaram, Caluire, France) instrument coupled with a mass spectrometer. The measurements were performed in synthetic air (30 mL min^{-1}) from 20 to 750 °C with a heating rate of 10 °C min^{-1}. Fourier transform infrared (FTIR) spectra were collected with a Nicolet NEXUS 670-FT spectrometer (Thermo Fisher Scientific, Waltham, MA, USA) with an ATR accessory. The content of C, H and N was determined by a standard combustion technique (Thermo Scientific FlashSmartTM 2000 Elemental analyzer). The content of P and Fe was measured by ICP-MS (Agilent 7900 equipped with an Ar burner, ORS 4 collision cell and orthogonal hyperbolic quadrupole mass analyser), 20 ppb indium solution was used as an internal standard. Prior the measurement, the samples were dissolved in the mixture of acids (12 mL of HCl, 4 mL of HNO_3, and 4 mL HF for 10 mg of sample) under microwave irradiation.

The removal of pollutants and linker release were quantified using a high-performance liquid chromatography (HPLC) Agilent 1260 Infinity II instrument with a diode array detector (DAD) and ASTRA C18-HE (partial size 3 µm, 50 x 3 mm) chromatographic column. In each case 10 µL of the sample was injected using an autosampler. The analyses were carried out at a constant temperature of 30 °C. A mixture of 0.02M phosphate buffer (PB) solution with pH 2.5 and acetonitrile (MeCN) was used as a mobile phase. The flow rate was 0.5 mL min^{-1} under isocratic elution for all analytes. The composition of the mobile phase for particular analytes, wavelengths used for the detection, time of analysis, limits of detection (LOD) and limits of quantification (LOQ) are specified in Table S1. The table also shows the parameters for H_2BBP(Ph) and H_2BBP(Ph-NMe_2), which were detected to see how much of the linker was released from the materials. The amount of H_2BBP(Ph-COOEt) released from ICR-14 was determined by ICP-MS to avoid the effect of potential linker hydrolysis providing a product with a different retention on the HPLC column. The LOQ of ICP-MS was 0.005 mg L^{-1} of phosphorus.

Table S1: Parameters of the HPLC determination of particular analytes.

Analyte	PB (vol.%)	MeCN (vol.%)	Detection wavelength (nm)	Time of analysis (min)	LOD (mg L^{-1})	LOQ (mg L^{-1})	Error of repeated analysis (RSD %)
DCF	40.0	60.0	276	3.5	0.02	0.07	< 3
SMX	70.0	30.0	270	3.5	0.01	0.05	< 4
CEP	85.0	15.0	261	3.5	0.05	0.20	< 3
$H_2BBP(Ph)$	77.5	22.5	200 or 272	9.0	0.05	0.10	< 8
$H_2BBP(Ph\text{-}NMe_2)$	80.0	20.0	200 or 276	9.0	0.05	0.10	< 5

Mathematical models for adsorption experiments

Collected data from adsorption experiments were evaluated using the follows equations:

Adsorbed amount of the pharmaceutics, q_e (mg g^{-1}), was calculated using (1):

$$q_e = \frac{(C_0 - C_e)\,V}{m} \qquad (1)$$

where C_0 (mg dm^{-3}) is initial concentration of the pollutant, C_e (mg dm^{-3}) represents equilibrium concentration of the pollutant obtained by HPLC, and m (g) and V (dm^3) stand for weight of the adsorbent and total volume of the pollutant solution, respectively.

The kinetic data of the adsorption were fitted with a pseudo-second order kinetic model with (2):

$$q_e = Q_{max}\,\frac{Q_{max}\,k_2\,t}{1+Q_{max}\,k_2\,t} \qquad (2)$$

where q_e (mg g^{-1}) is the concentration of the pollutant adsorbed in the defined time, Q_{max} (mg g^{-1}) is the concentration of the pollutant adsorbed in the equilibrium point. Pseudo-second order rate constant and time are in (2) marked as k_2 (g mg^{-1} min^{-1}) and t (min), respectively. [S1]

Adsorption isotherms were described using Langmuir (3) and Freundlich (4) models:

$$q_e = Q_{max}\,\frac{K_L\,C_e}{1+K_L\,C_e} \qquad (3)$$

where q_e is the adsorbed amount of the pollutant at defined concentration calculated by (1), Q_{max} is the Langmuir maximum sorption capacity (mg g^{-1}) representing the saturation plateau; C_e represents the equilibrium concentration measured by HPLC (mg dm^{-3}); and K_L stands for the Langmuir constant (dm^3 mg^{-1}) quantifying the affinity of the adsorbate to the adsorbent. [S2]

$$q_e = K_F C_e^{\frac{1}{n}} \qquad (4)$$

where K_F ([(mg g^{-1}) (mg dm^{-3})$^{-n}$] represents the Freundlich constant and *n* is the parameter of surface heterogeneity, which is related to the sorption capacity. [S2]

Since the final isotherm is S-shaped in the case of sulfamethoxazole adsorption by ICR-7, a modified Sips isotherm, which is a combination of Langmuir and Freundlich isotherm, was used for data fitting. Its mathematical expression is given by (5):

$$q_e = Q_{max} \frac{K_S\, C_e^n}{1+K_S\, C_e^n} \quad (5)$$

where K_S and *n* stand for Sips constant parameters and their meaning is derived from Langmuir and Freundlich model. [S3]

Supplementary Figures and Data

Elemental analysis

Table S2: Elemental composition of the ICR MOFs determined by CHN analysis and ICP-MS. The values in brackets show the theoretical composition calculated based on the empirical formula $C_{72}H_{54}Fe_2O_{12}P_6$ for ICR-7, $C_{84}H_{84}Fe_2O_{12}N_6P_6$ for ICR-8 and $C_{78}H_{78}Fe_2O_{24}P_6$ for ICR-14, respectively.

	**C / wt %**	**H / wt %**	**N / wt %**	**P / wt %**	**Fe / wt %**
**ICR-7**	60.94 (61.39)	3.74 (3.86)	-	4.37 (13.19)	7.54 (7.93)
**ICR-8**	50.12 (60.52)	4.25 (5.08)	1.95 (5.04)	8.63 (11.15)	7.67 (6.70)
**ICR-14**	52.08 (55.21)	3.84 (4.63)	0.21 (0.00)	5.13 (10.95)	5.58 (6.58)

Structural analysis

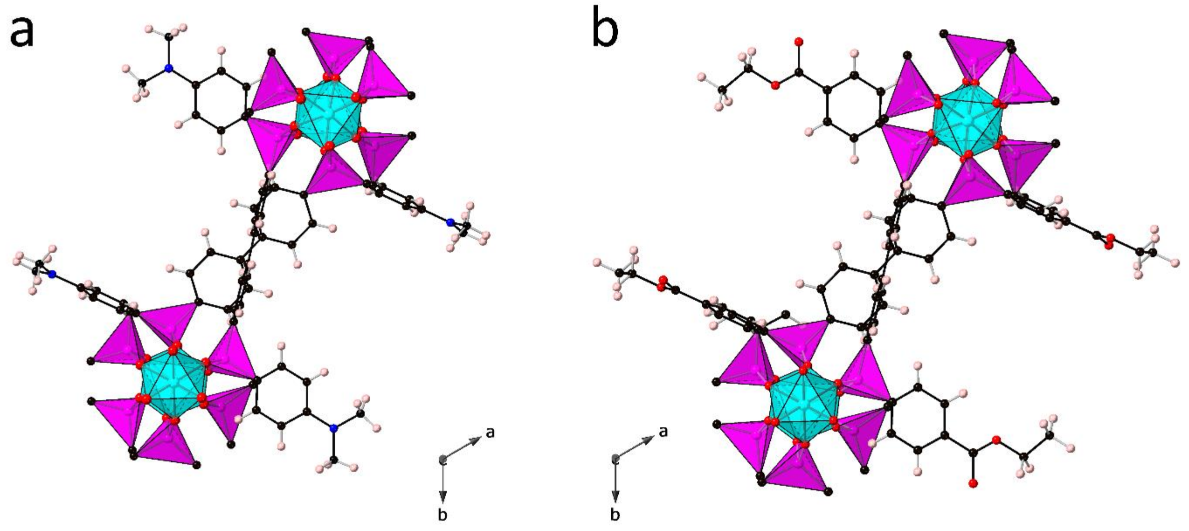

Figure S1: The top view on the 1D chains in ICR-8 (a) and ICR-14 (b). Iron octahedra are light blue, phosphinate tetrahedra are magenta; elements are colour-coded as follows: Fe (light blue), P (magenta), O (red), C (black), N (blue) and H (white).

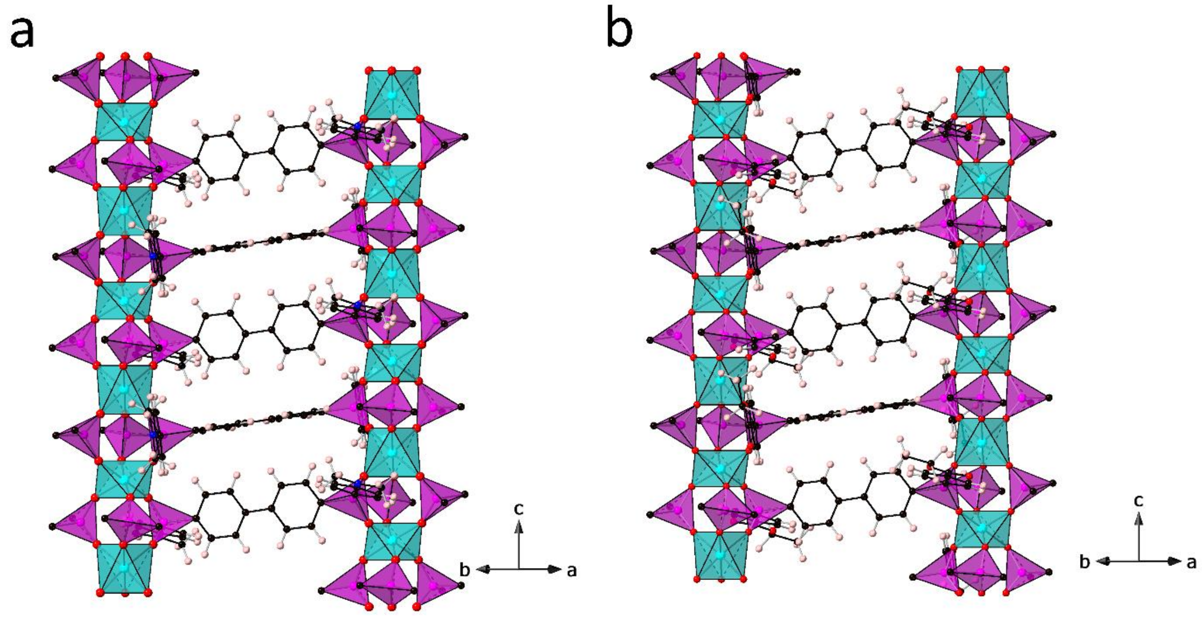

Figure S2: The side view of the 1D chains composed of octahedrally coordinated iron atoms (blue polyhedron) and tetrahedrons of phosphorus atoms surrounded by two oxygen and two carbon atoms in the structure of ICR-8 (a) and ICR-14 (b). Iron octahedra are light blue, phosphinate tetrahedra are magenta; elements are colour-coded as follows: Fe (light blue), P (magenta), O (red), C (black), N (blue) and H (white).

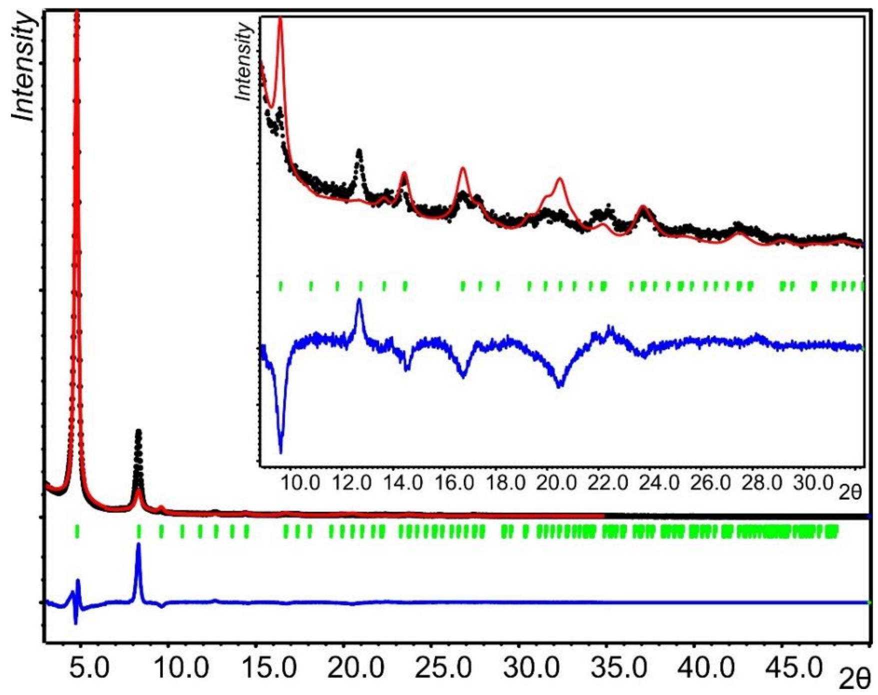

Figure S3: Rietveld fit of ICR-8. Measured powder XRD pattern (black), theoretical pattern calculated from the manually created crystal structure (red), Bragg's positions (green) difference between measured and calculated profile (blue) are depicted.

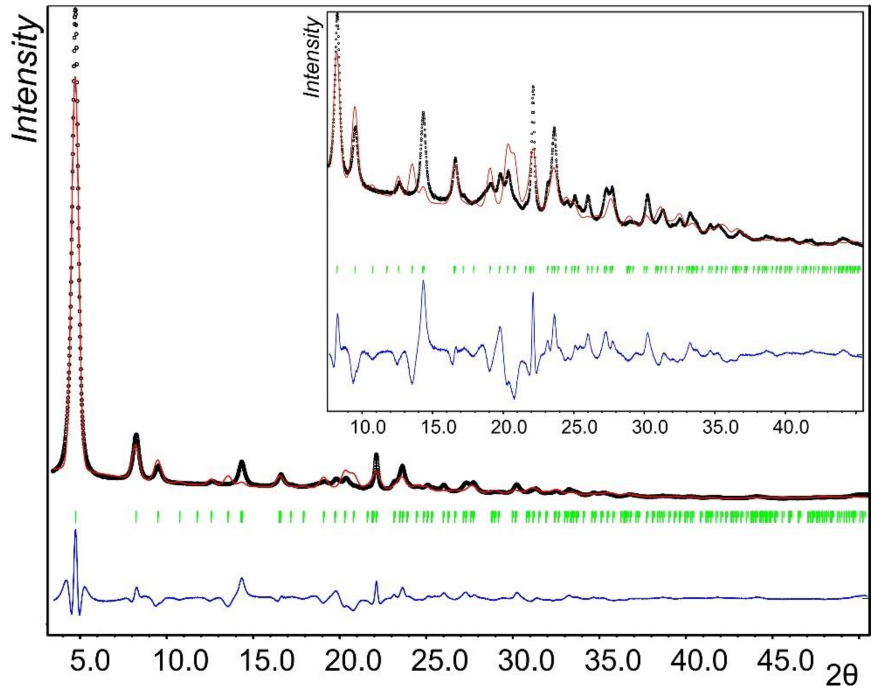

Figure S4: Rietveld fit of ICR-14. Measured powder XRD pattern (black), theoretical pattern calculated from the manually created crystal structure (red), Bragg's positions (green) difference between measured and calculated profile (blue) are depicted.

Infrared spectra

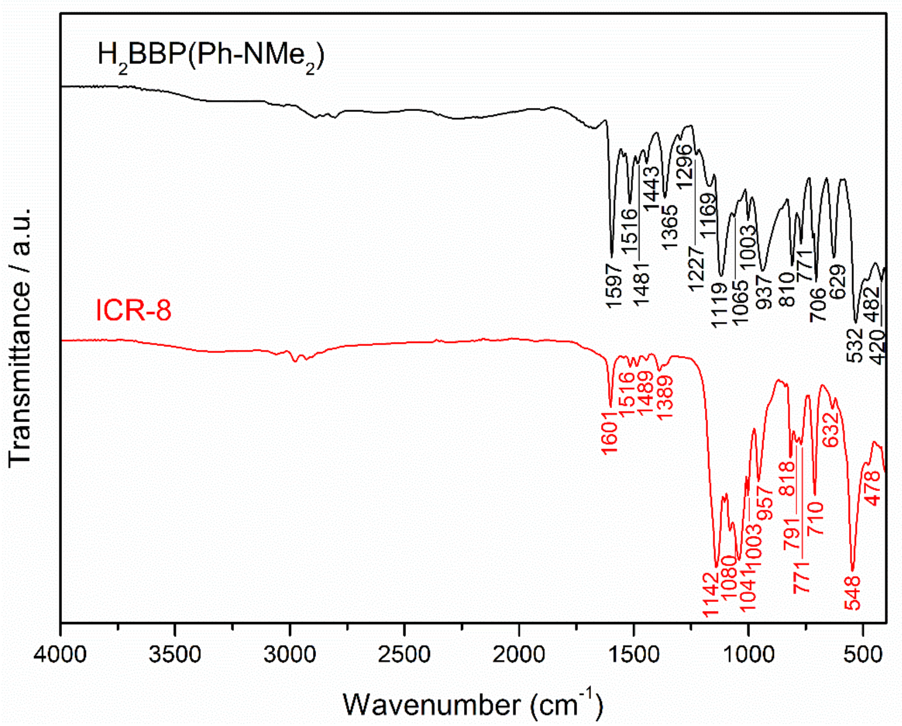

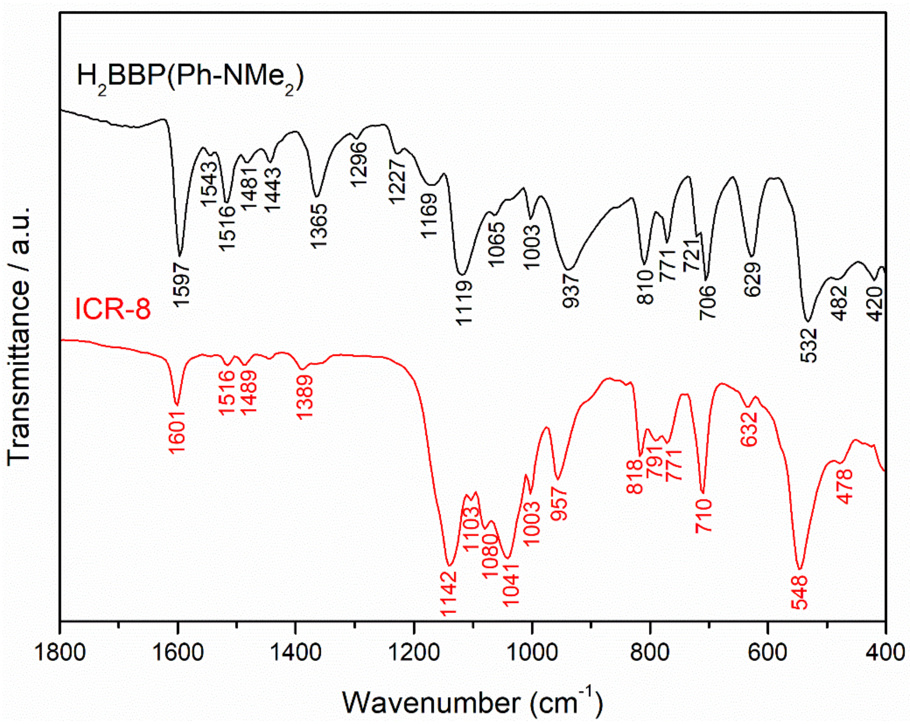

Figure S5: Infrared spectra of ICR-8 (below) and the precursor molecule $H_2BBP(NMe_2)$ (top).

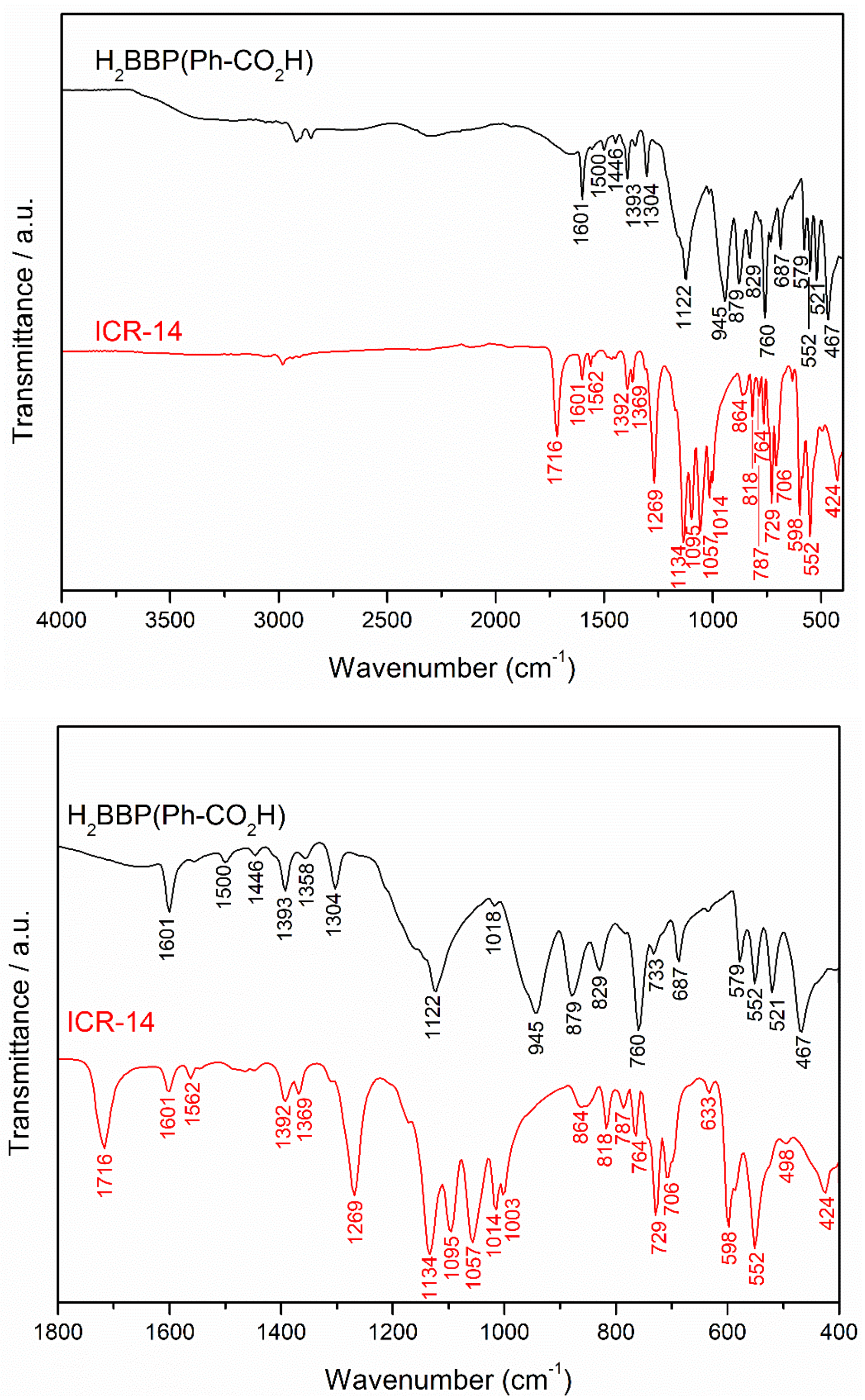

Figure S6: Infrared spectra of ICR-14 (below) and the precursor molecule $H_2BBP(Ph\text{-}CO_2H)$ (top).

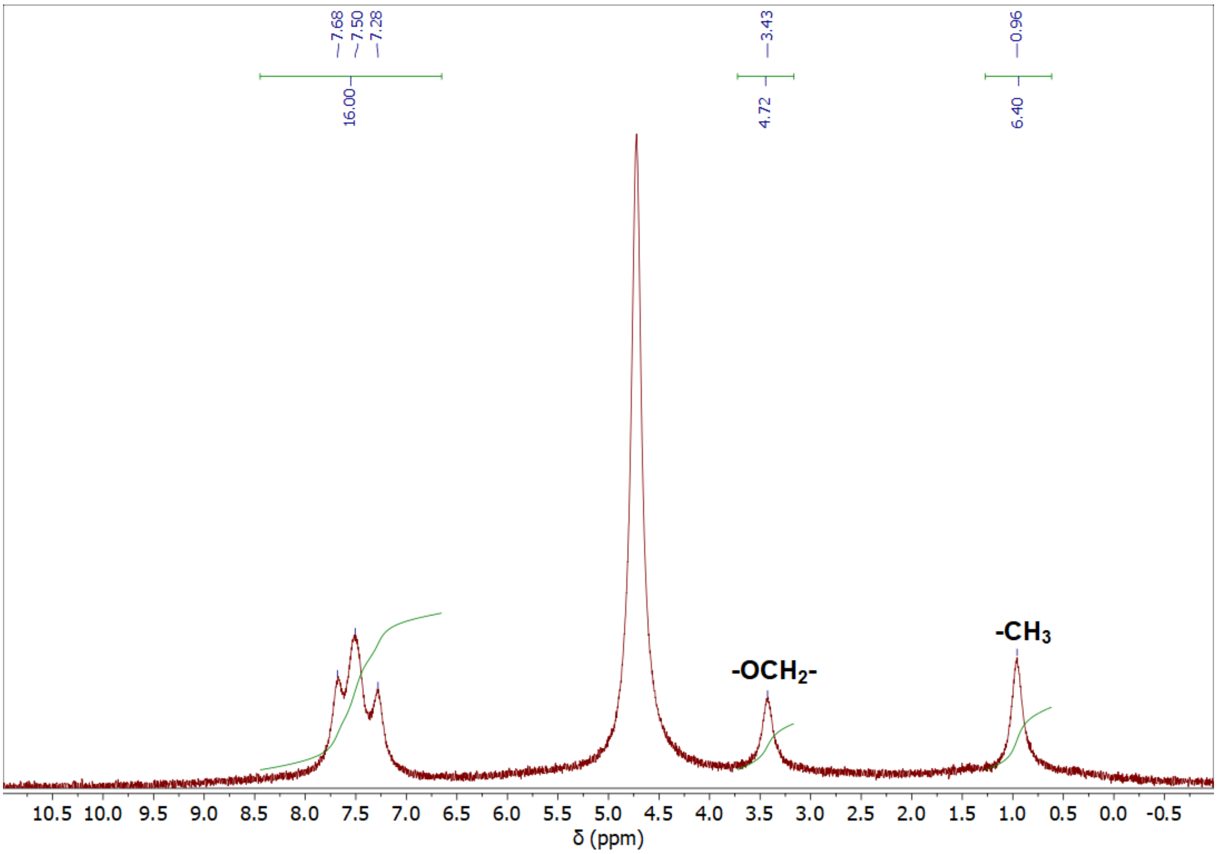

Figure S7: ^{1}H NMR spectrum of ICR-14 dissolved in 0.1M deuterated KOH solution.

Thermogravimetric analysis

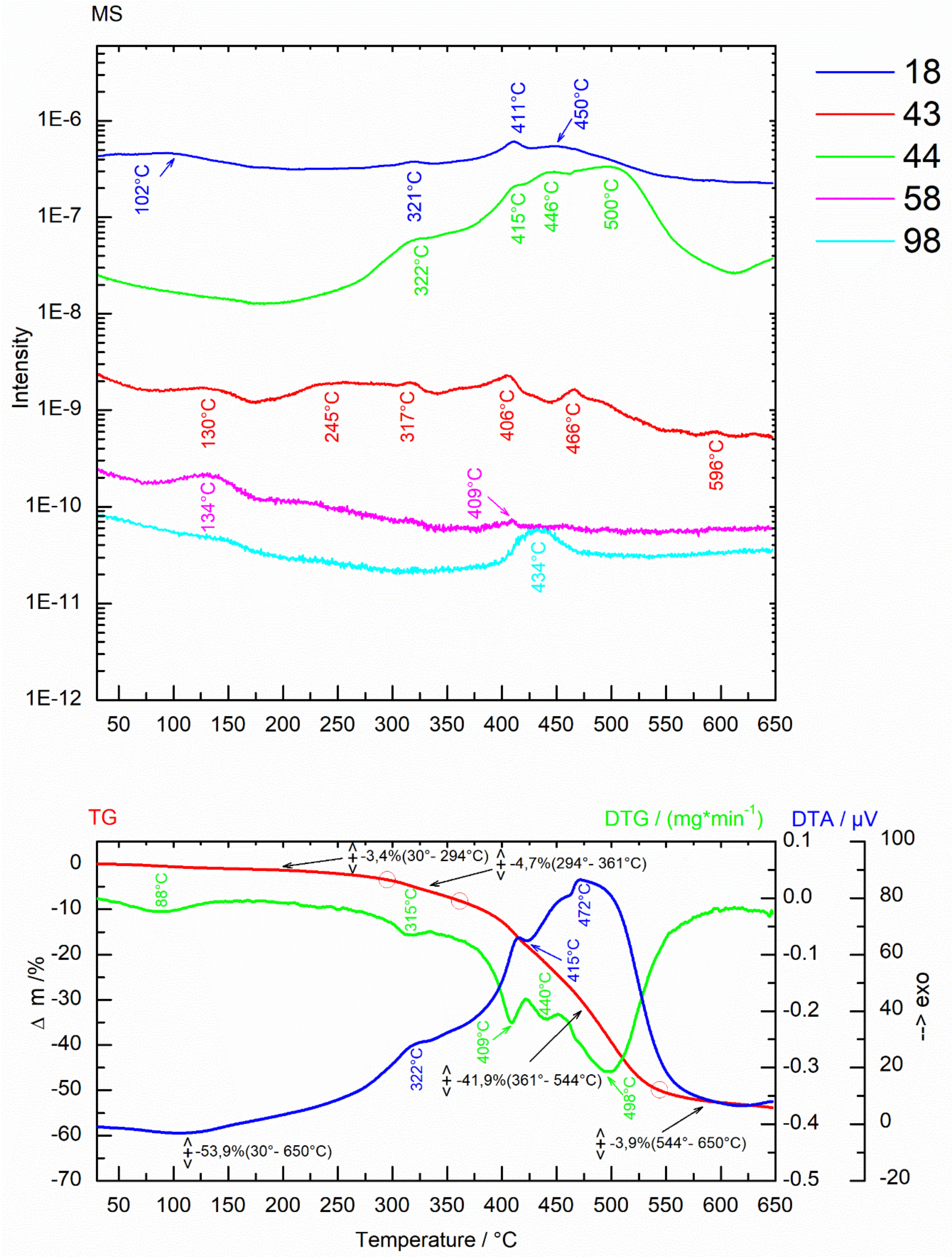

Figure S8: TGA/DTA curves (bottom) and the evolution of gases (top) for ICR-8 in air; m/z = m/z = 18 – H_2O, m/z = 44 – CO_2, m/z = 43 and 58 – acetone, and m/z = 98 – H_3PO_4.

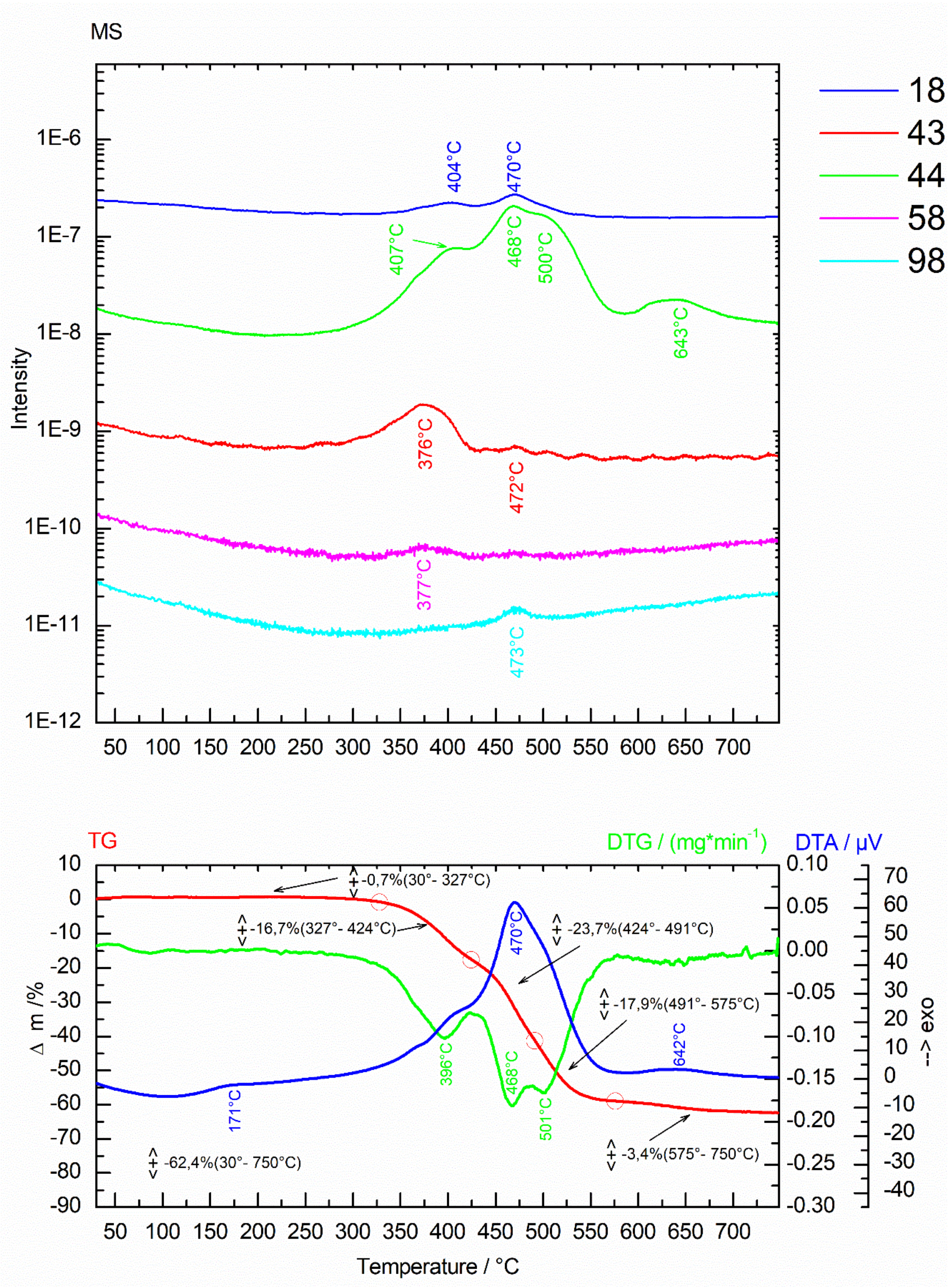

Figure S9: TGA/DTA curves (bottom) and the evolution of gases (top) for ICR-14 in air; m/z = m/z = 18 – H_2O, m/z = 44 – CO_2, m/z = 43 and 58 – acetone, and m/z = 98 – H_3PO_4.

Porosity determination

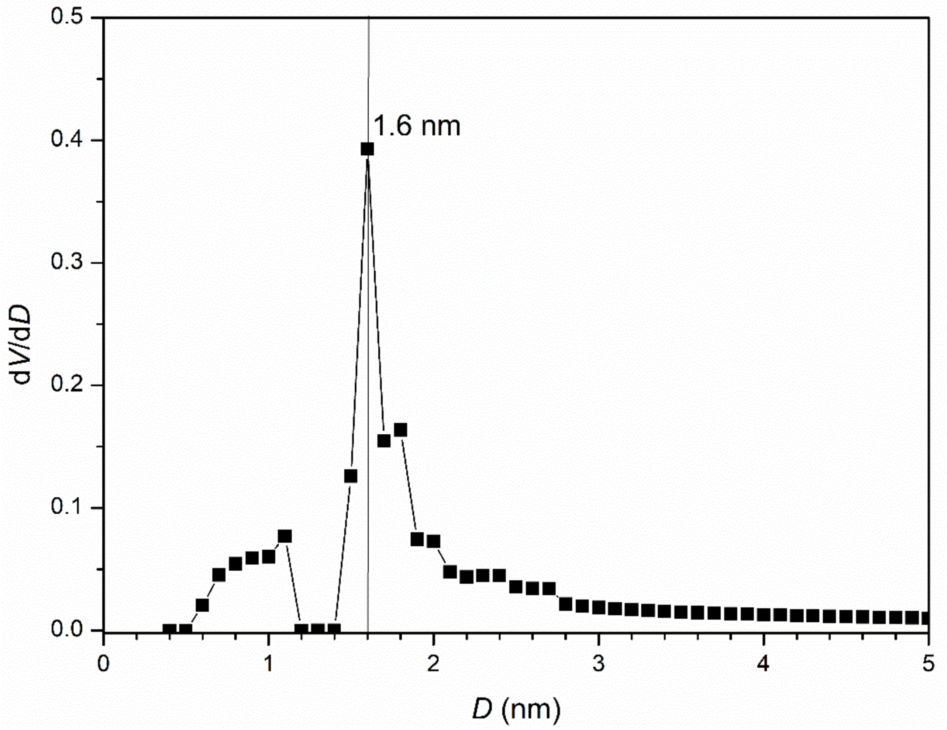

Figure S10: Pore size distribution for ICR-7 calculated by DFT method.

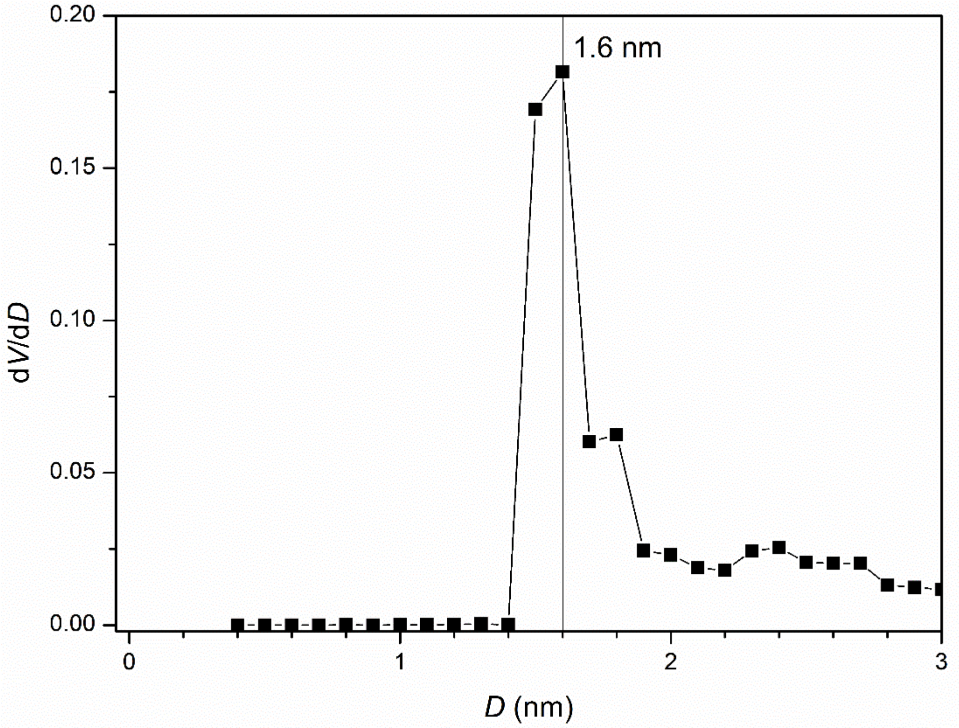

Figure S11: Pore size distribution for ICR-8 calculated by DFT method.

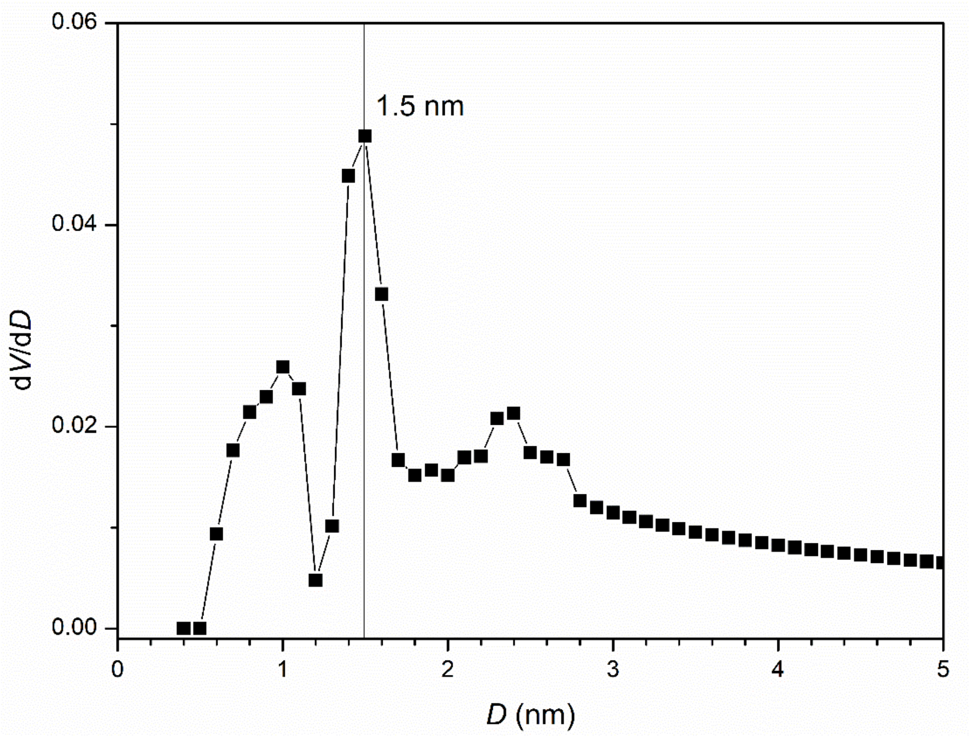

Figure S12: Pore size distribution for ICR-14 calculated by DFT method.

Adsorption of pharmaceutical pollutants

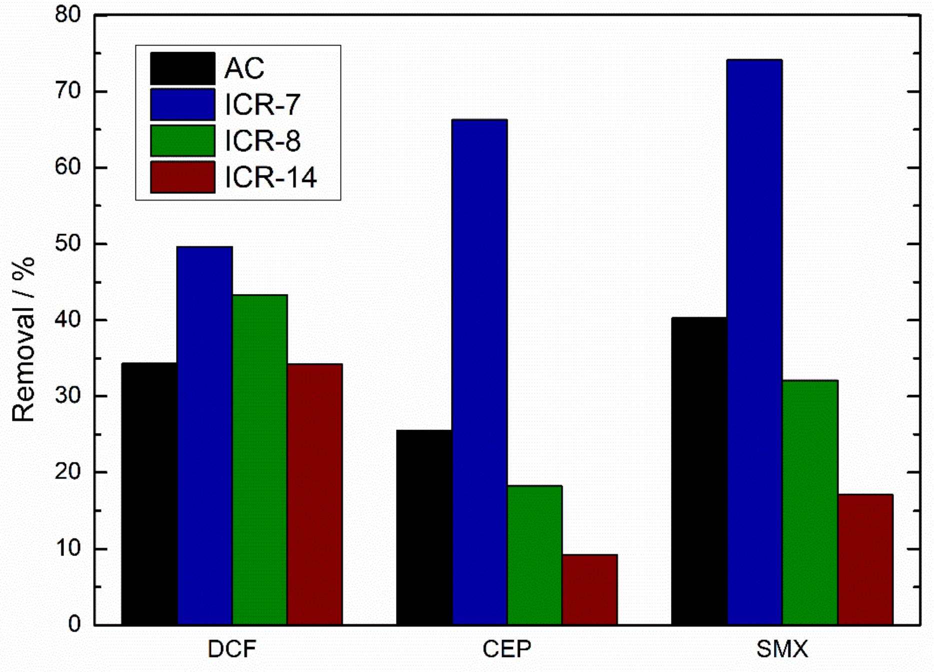

Figure S13: Comparison of the removal efficiency of the tested adsorbents for particular pharmaceutical pollutants; the percentage of the initial amount of the pollutant which was captured after 24 h from a 100 mg L^{-1} solution by 5 mg of an adsorbent is depicted.

Stability studies

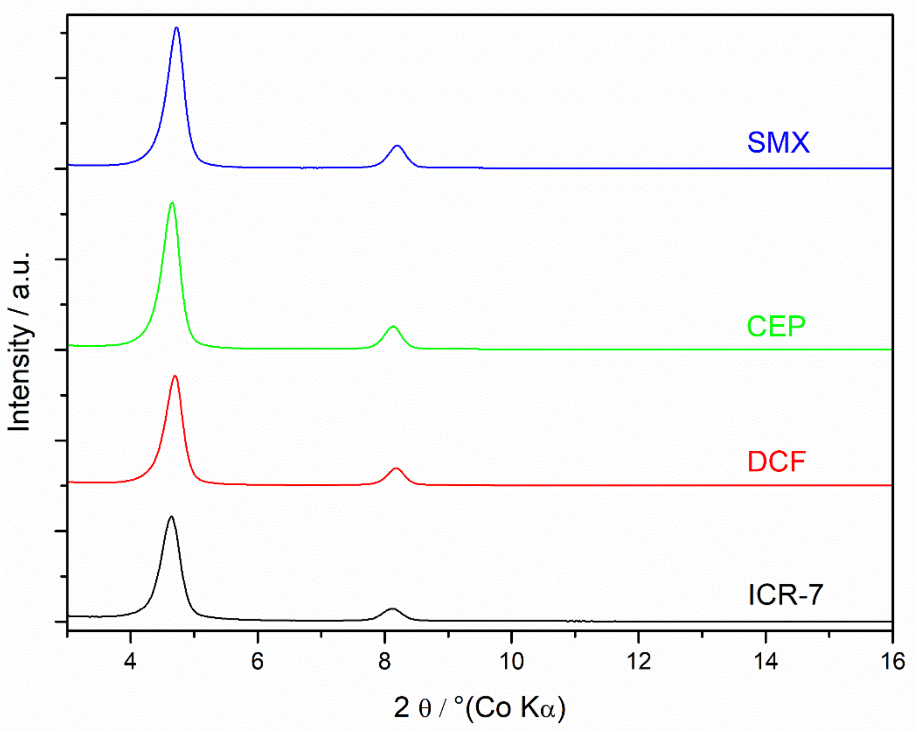

Figure S14: Powder diffraction patterns of as-synthetized ICR-7 (black) and ICR-7 after the adsorption of DCF (red), CEP (green), and SMX (blue).

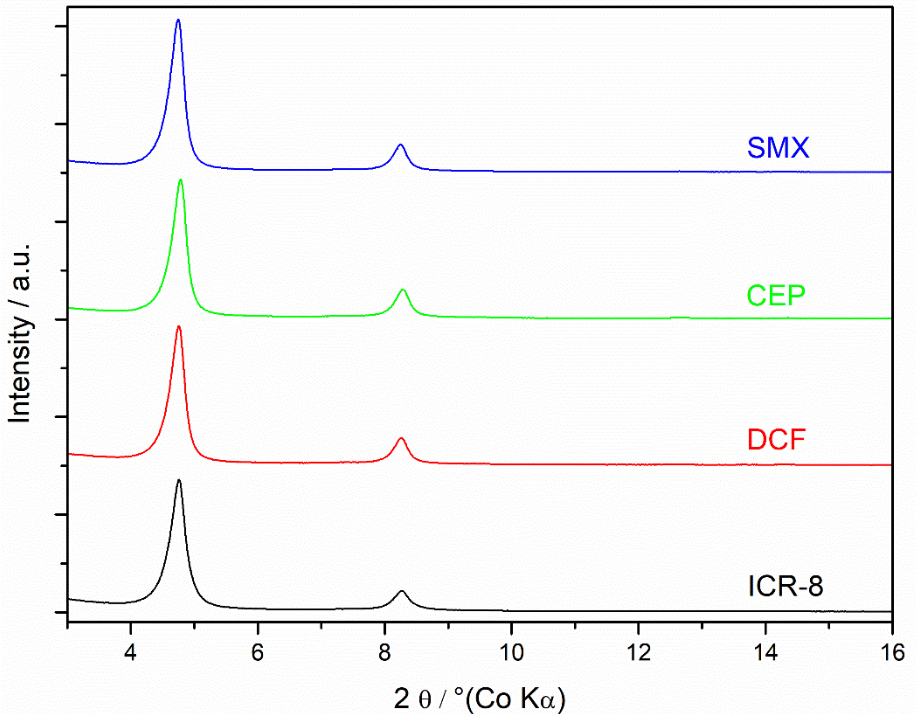

Figure S15: Powder diffraction patterns of as-synthetized ICR-8 (black) and ICR-8 after the adsorption of DCF (red), CEP (green), and SMX (blue).

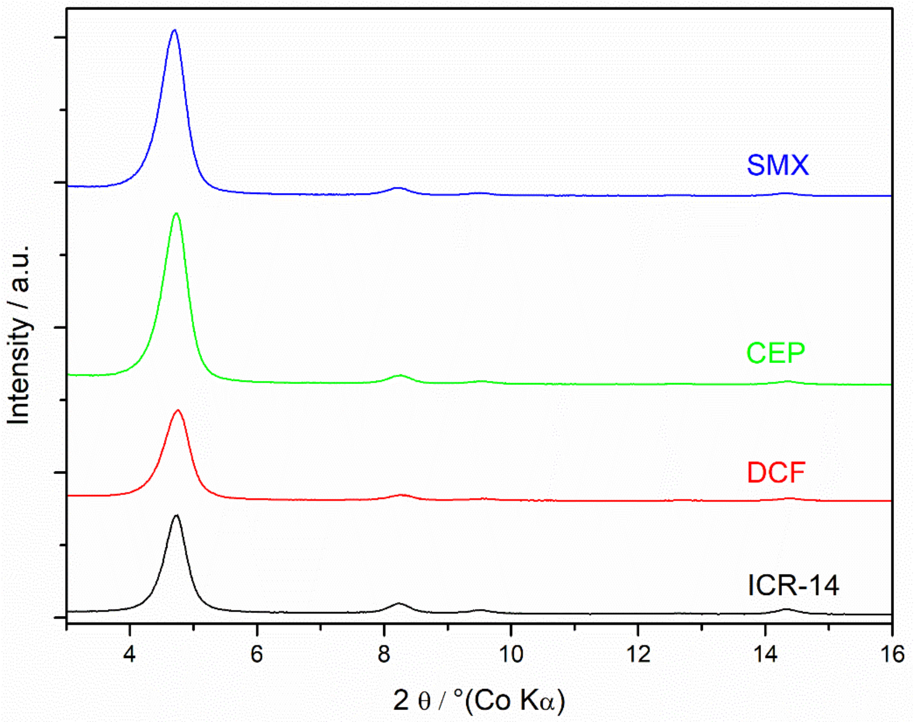

Figure S16: Powder diffraction patterns of as-synthetized ICR-14 (black) and ICR-14 after the adsorption of DCF (red), CEP (green), and SMX (blue).

Table S3: Specific surface areas of the as-prepared ICR MOFs and the materials after adsorption of pollutants; BET specific surface areas in $m^2\ g^{-1}$ are given.

**Sample**	**As prepared**	**DCF**	**CEP**	**SMX**
**ICR-7**	927	344	358	512
**ICR-8**	677	350	408	444
**ICR-14**	411	344	401	484

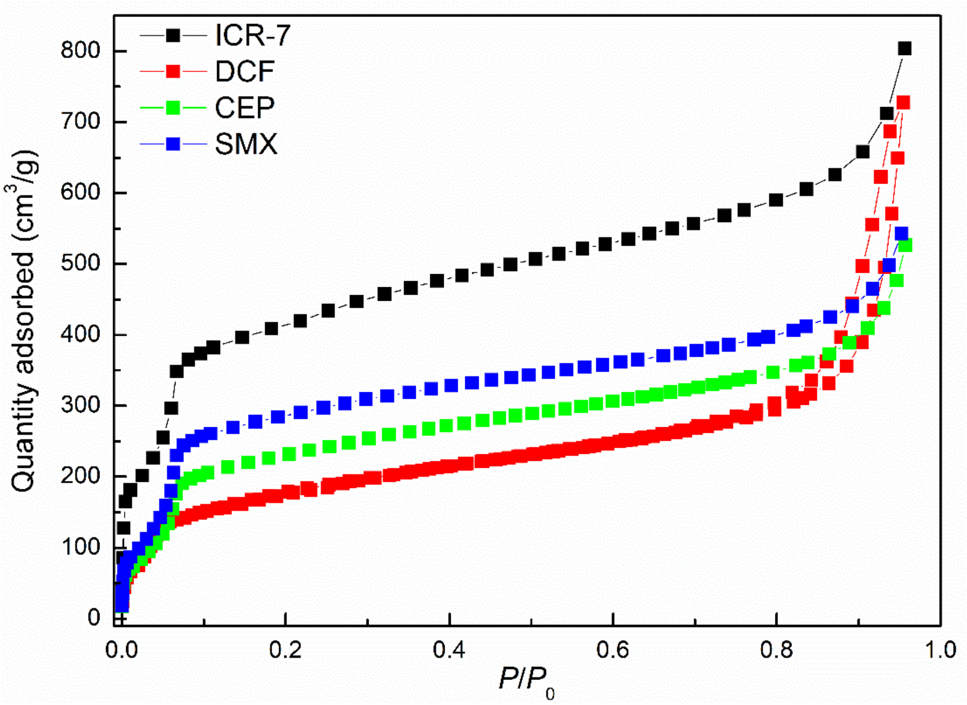

Figure S17: Adsorption isotherms of as-prepared ICR-7 (black) and ICR-7 after the adsorption and desorption of DCF (red), CEP (green), and SMX (blue). Measured using Ar (87 K) adsorbate.

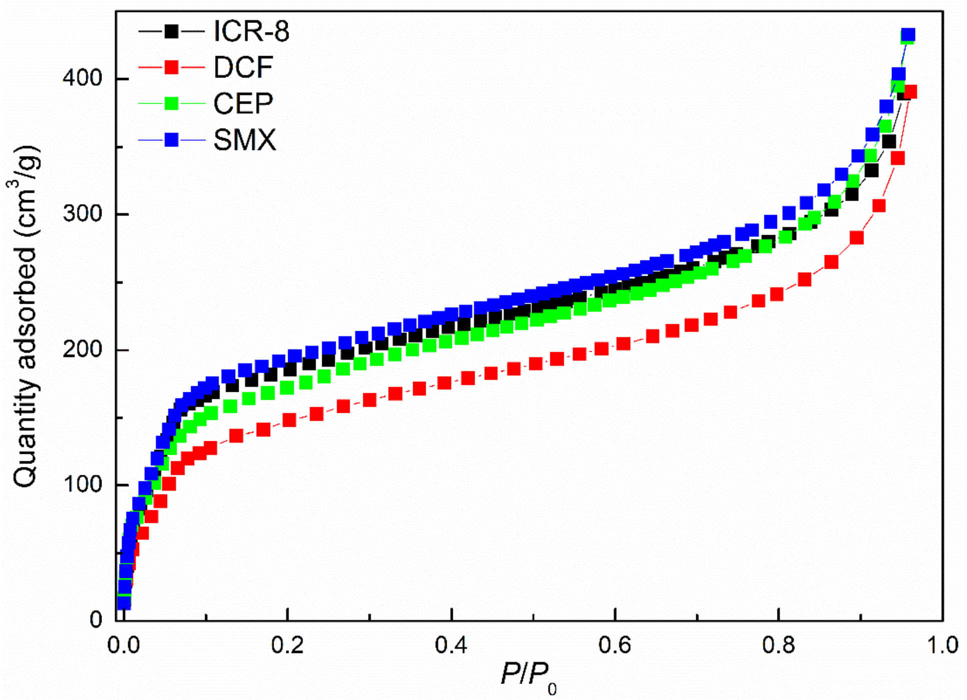

Figure S18: Adsorption isotherms of as-prepared ICR-8 (black) and ICR-8 after the adsorption and desorption of DCF (red), CEP (green), and SMX (blue). Measured using Ar (87 K) adsorbate.

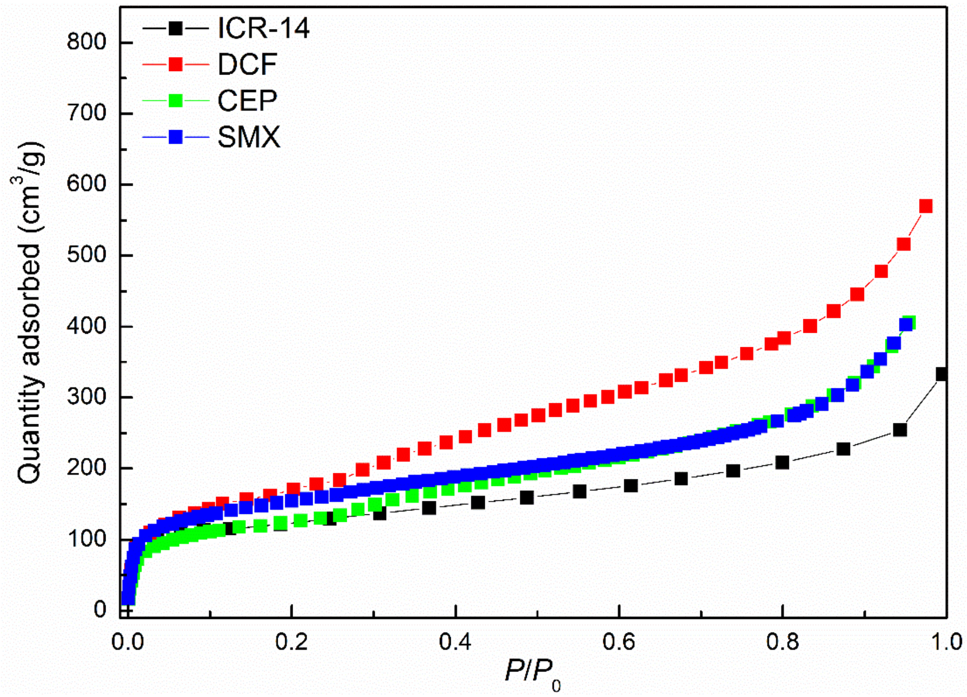

Figure S19: Adsorption isotherms of as-prepared ICR-14 (black) and ICR-14 after the adsorption and desorption of DCF (red), CEP (green), and SMX (blue). Measured using Ar (87 K) adsorbate.

Chemical analytical data for MOF precursors

^{1}H NMR spectrum of methyl 4-(*N*,*N*-dimethylamino)phenylphosphinate.

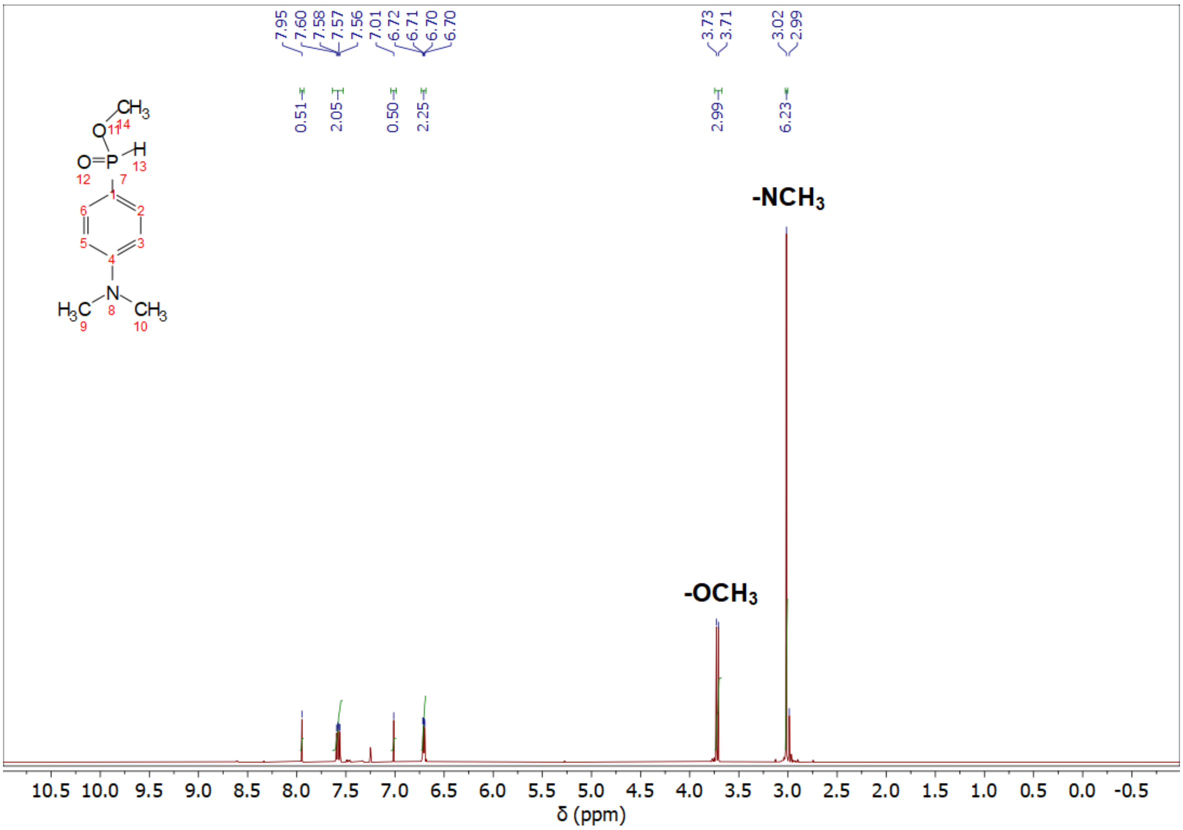

^{31}P NMR spectrum of methyl 4-(*N*,*N*-dimethylamino)phenylphosphinate.

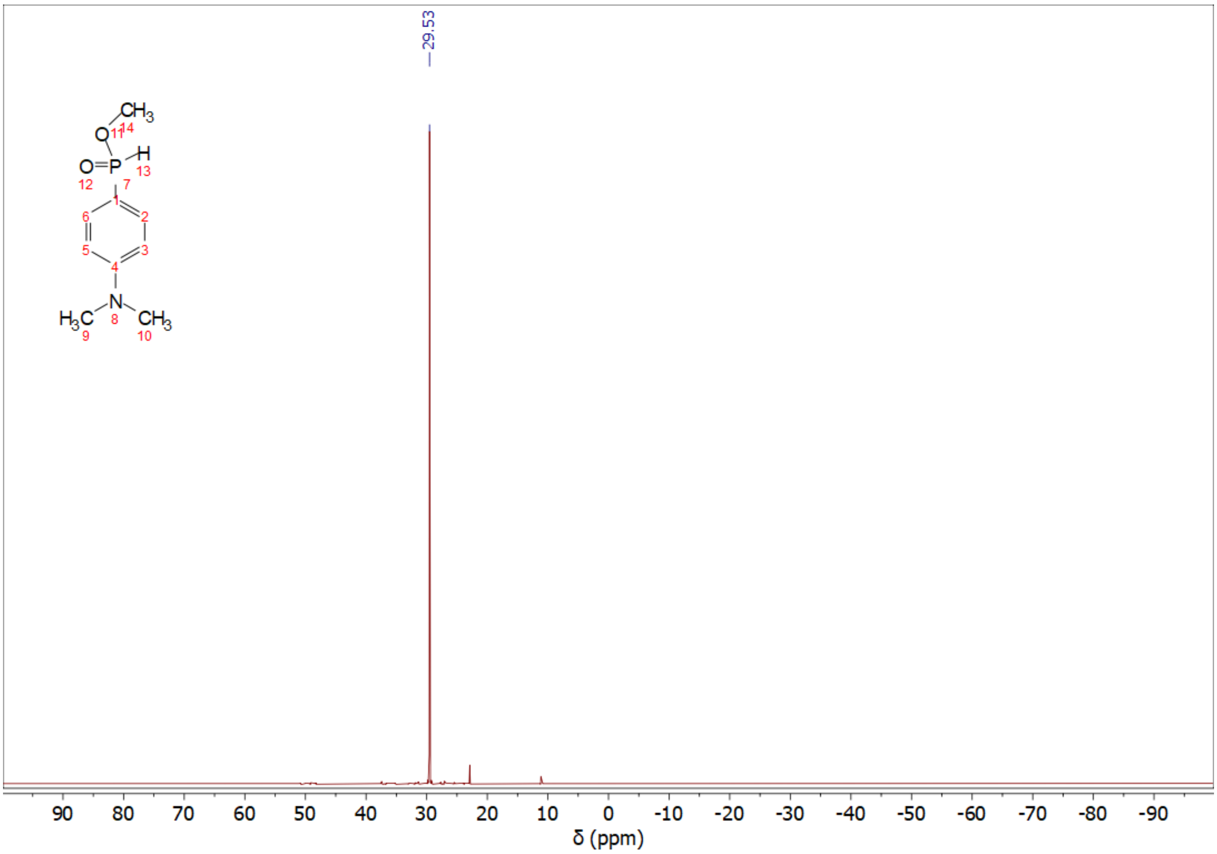

^{1}H NMR spectrum of dimethyl biphenyl-4,4ˈ-bis(4-(*N*,*N*-dimethylamino)phenylphosphinate).

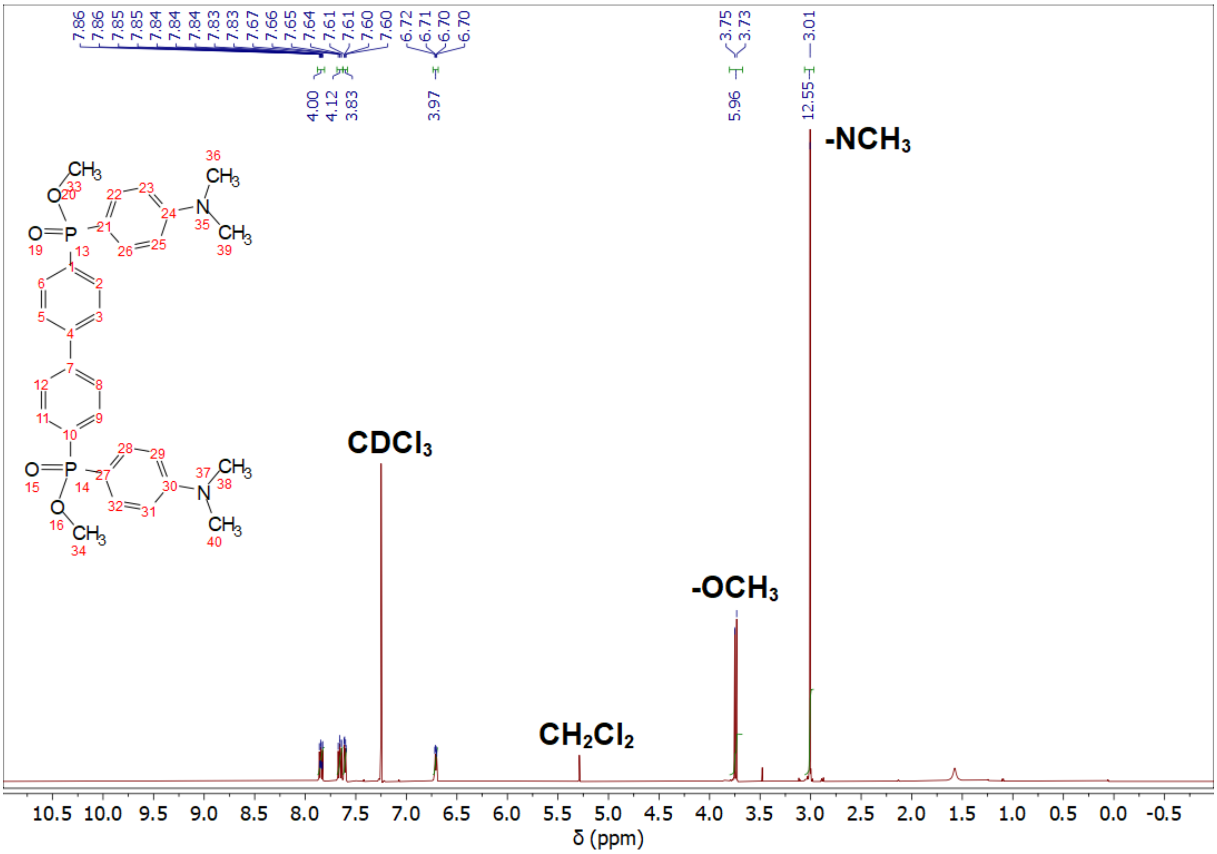

^{31}P NMR spectrum of dimethyl biphenyl-4,4ˈ-bis(4-(*N*,*N*-dimethylamino)phenylphosphinate).

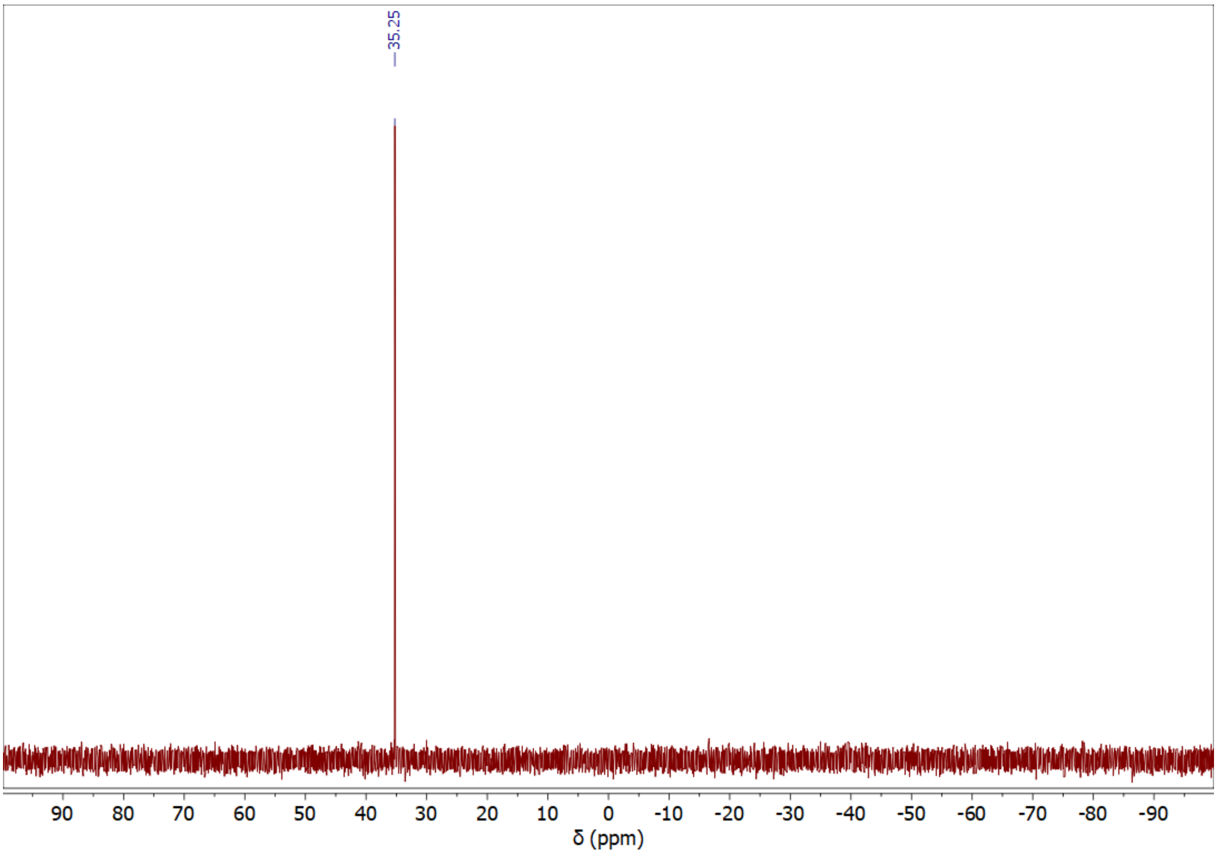

^{1}H NMR spectrum of biphenyl-4,4ˈ-bis(4-(*N*,*N*-dimethylamino)phenylphosphinic acid).

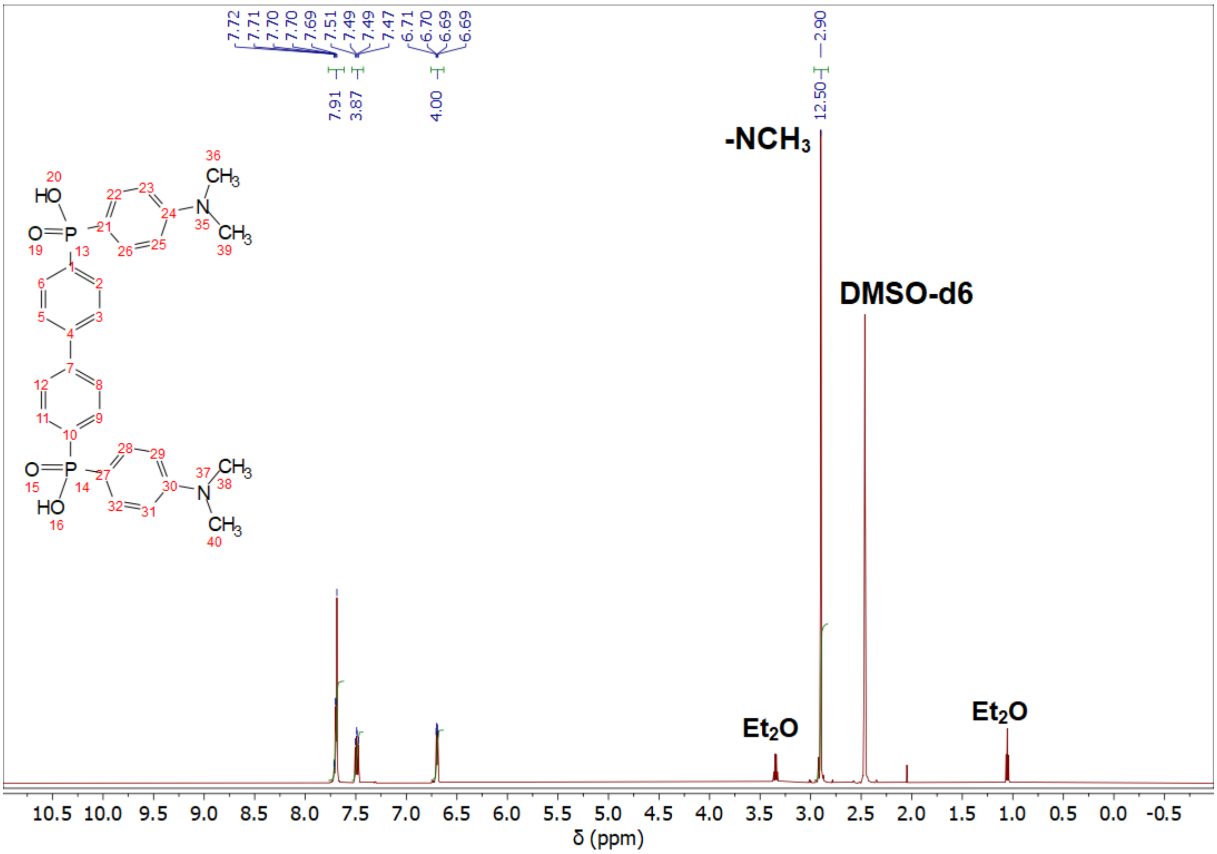

^{31}P NMR spectrum of biphenyl-4,4ˈ-bis(4-(*N*,*N*-dimethylamino)phenylphosphinic acid).

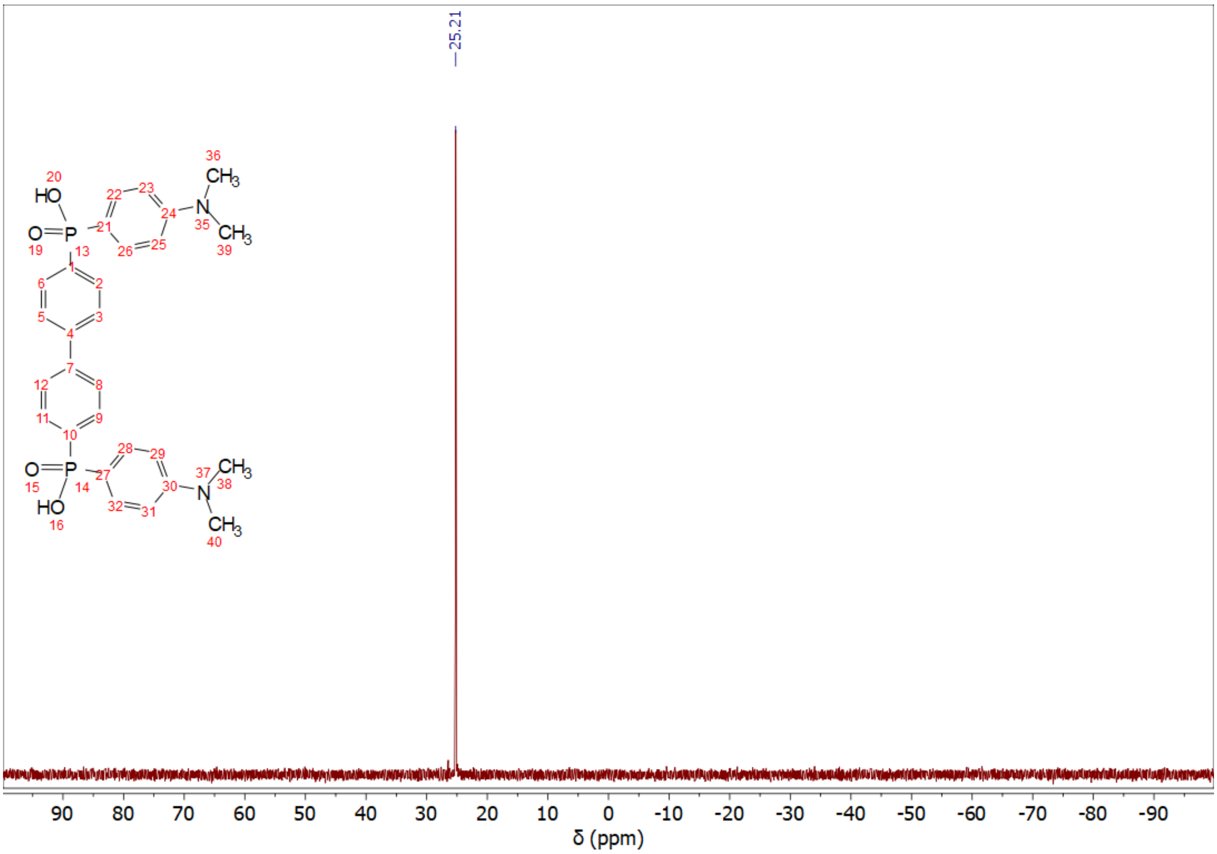

^{1}H NMR spectrum of dimethyl biphenyl-4,4'-diphosphinate.

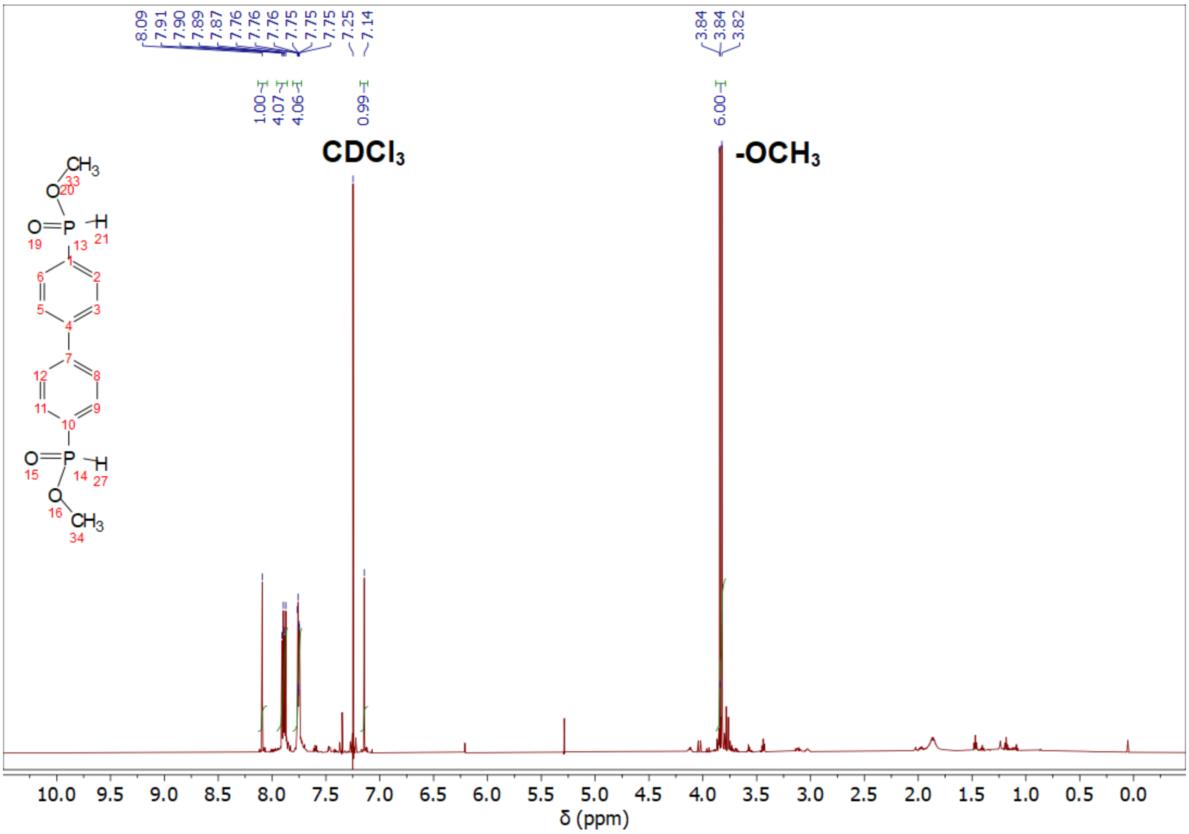

^{31}P NMR spectrum of dimethyl biphenyl-4,4'-diphosphinate.

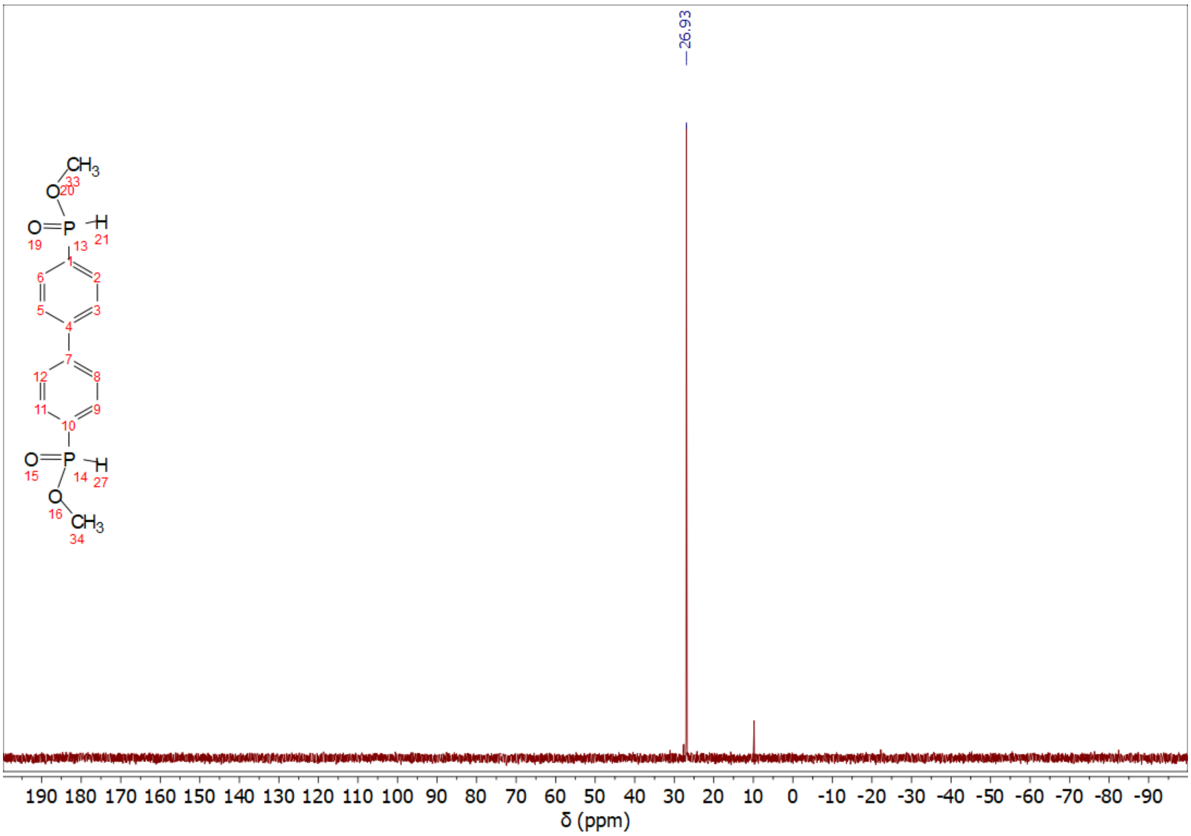

^1H NMR spectrum of dimethyl biphenyl-4,4'-bis(4-methoxycabonylphenylphosphinate).

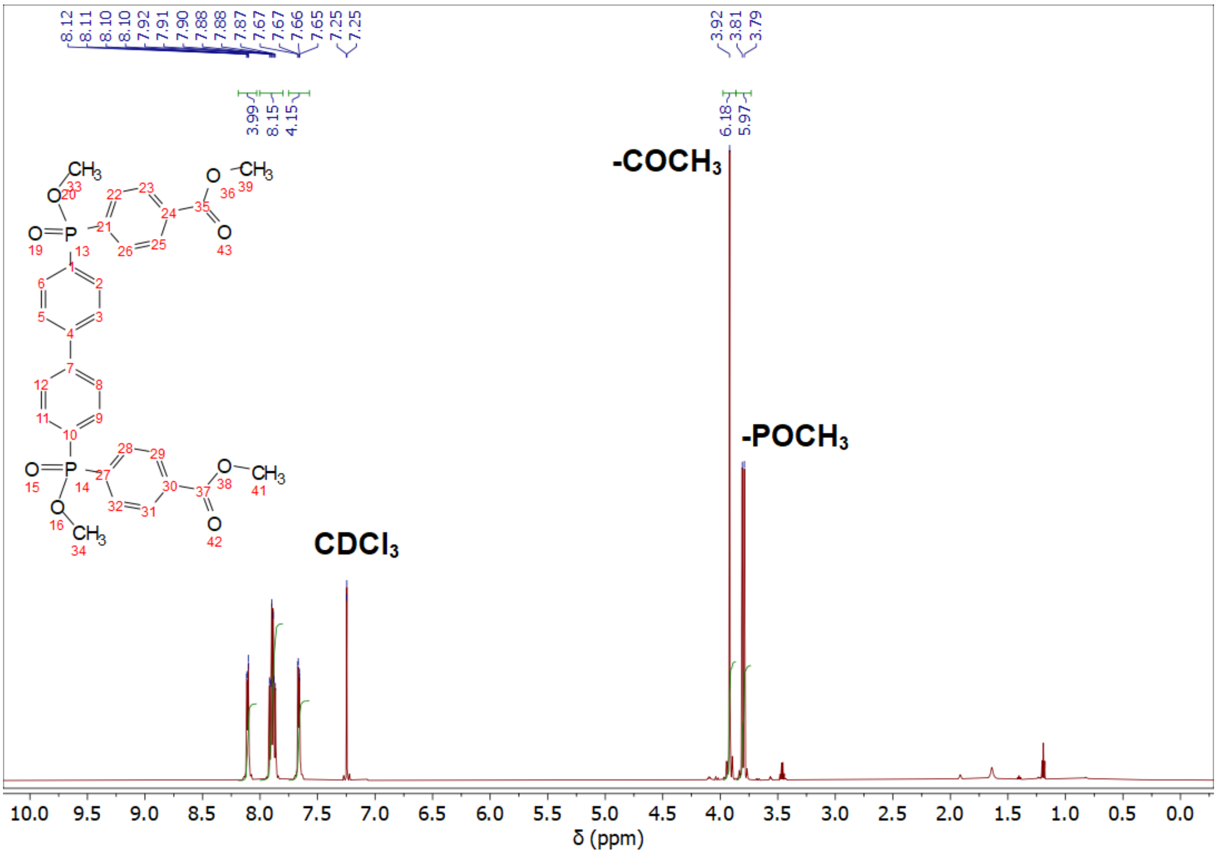

^{31}P NMR spectrum of dimethyl biphenyl-4,4'-bis(4-methoxycabonylphenylphosphinate).

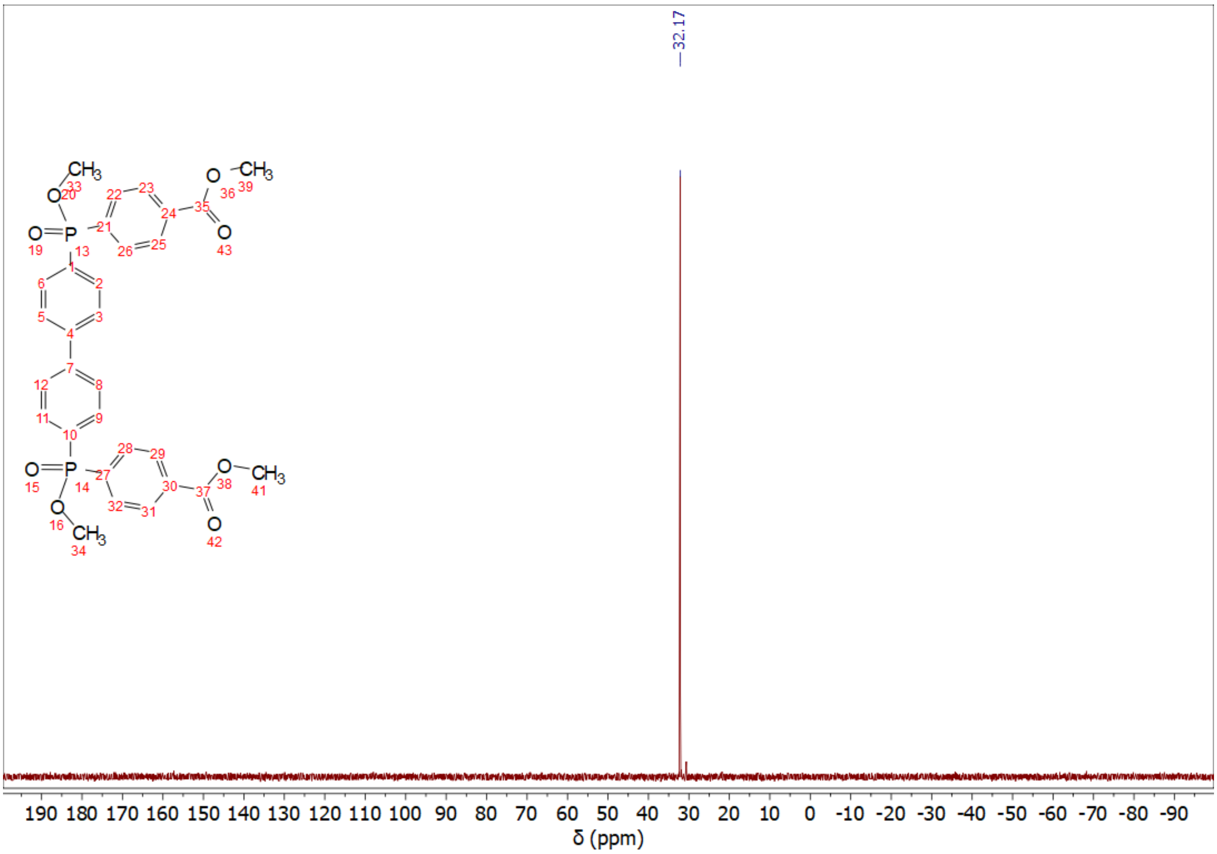

^{13}C NMR spectrum of dimethyl biphenyl-4,4'-bis(4-methoxycabonylphenylphosphinate).

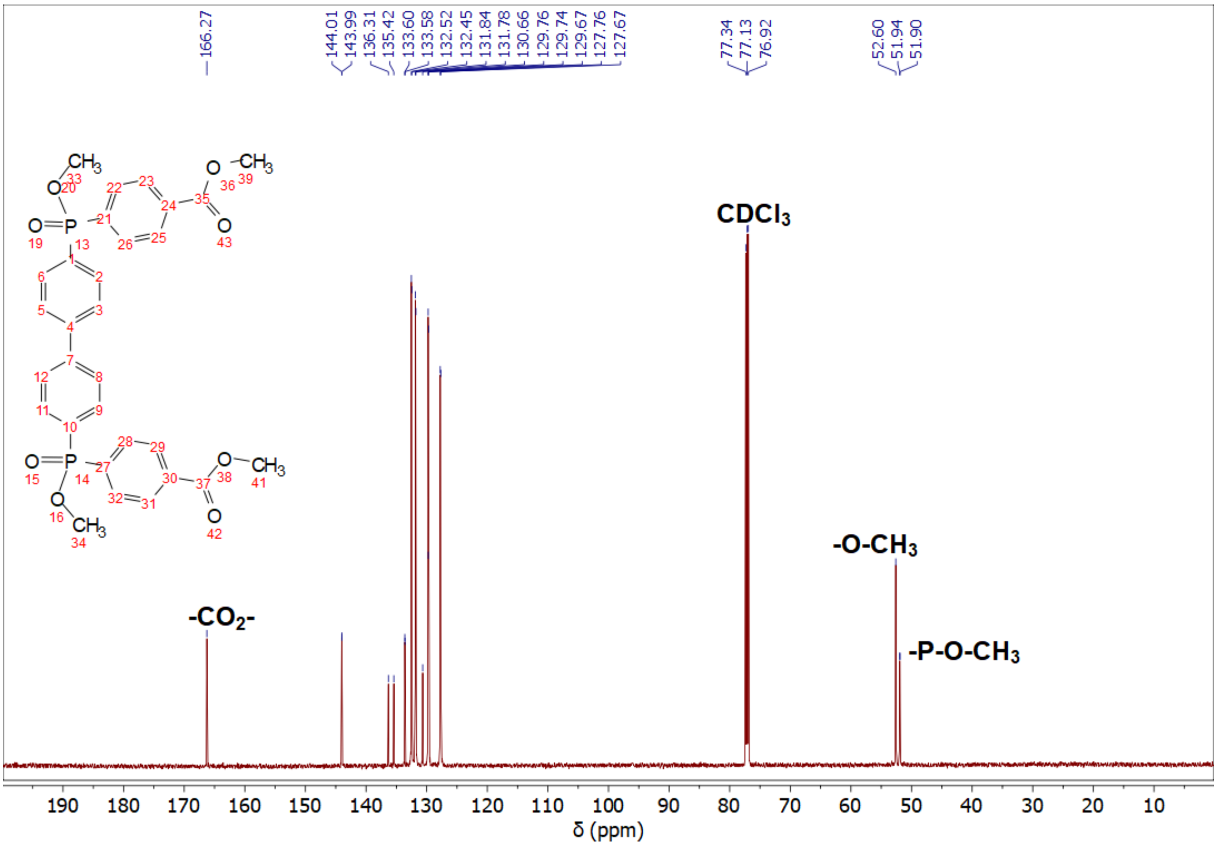

^{1}H NMR spectrum of biphenyl-4,4'-bis(4-methoxycabonylphenylphosphinic acid).

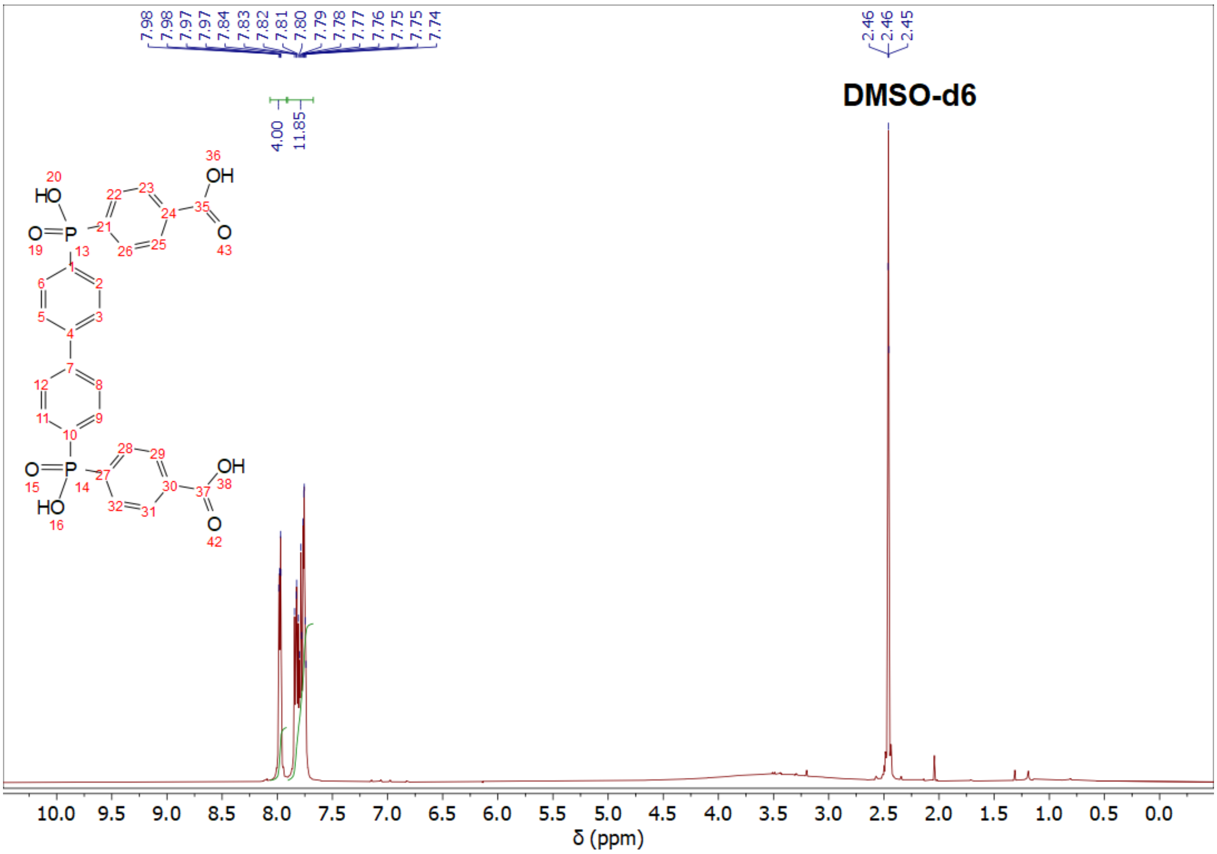

^{31}P NMR spectrum of biphenyl-4,4'-bis(4-methoxycabonylphenylphosphinic acid).

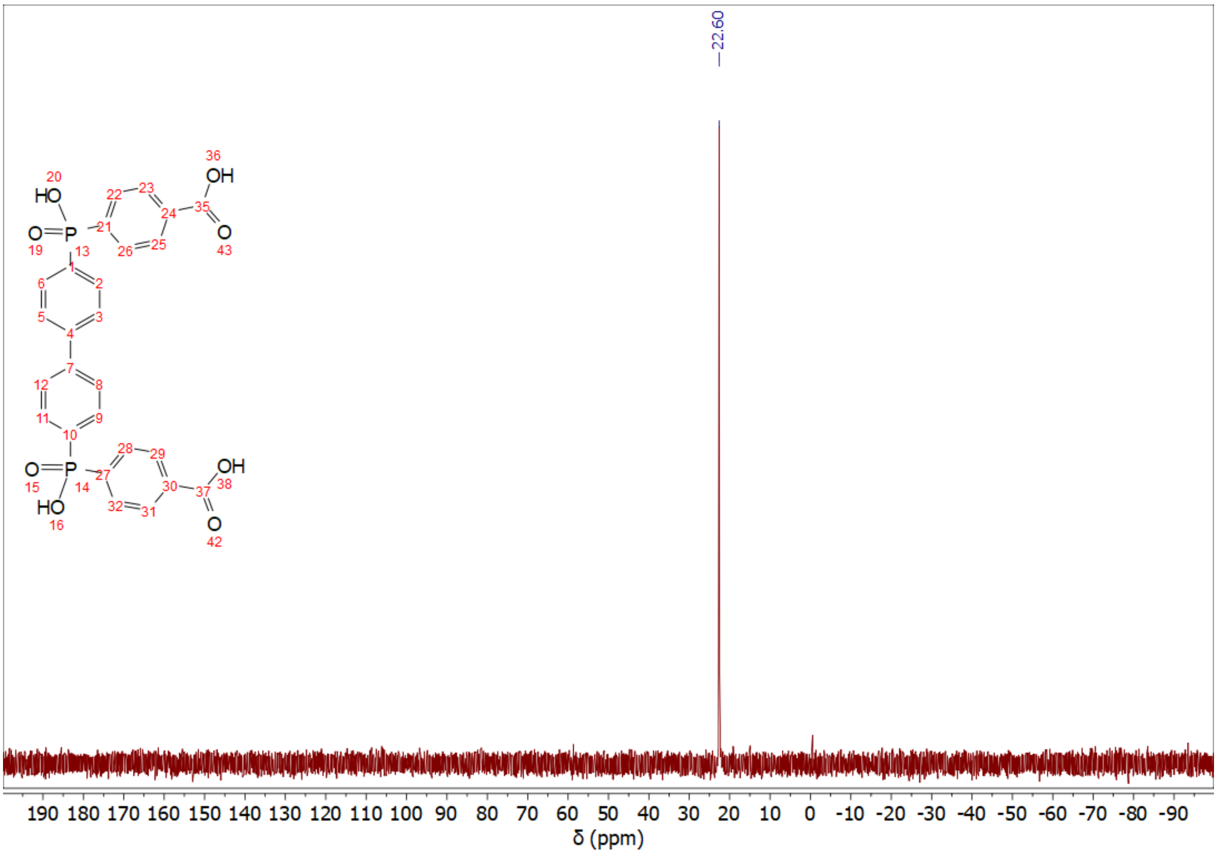

^{13}C NMR spectrum of biphenyl-4,4'-bis(4-methoxycabonylphenylphosphinic acid).

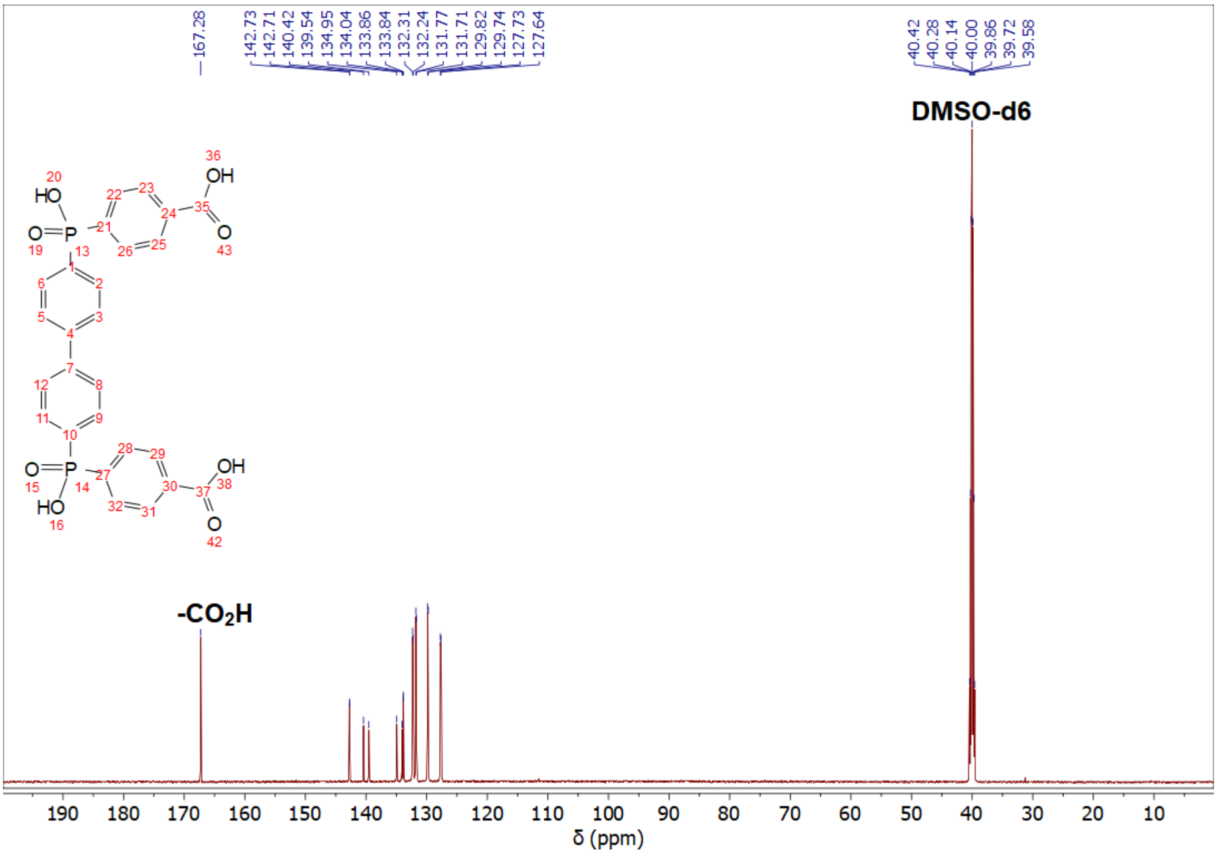

References:

[S1] J.–P. Simonin, On the comparison of pseudo-first order andpseudo-second order rate laws in the modelling of adsorption kinetics, *Chem. Eng. J*. **2016**, *300*, 254–263, https://doi.org/10.1016/j.cej.2016.04.079.

[S2] M. Ateia, D. E. Helbling, W. R. Dichtel, Best Practices for Evaluating New Materials as Adsorbents for Water Treatment, *ACS Mater. Lett.* **2020**, *2*, 1532–1544, https://doi.org/10.1021/acsmaterialslett.0c00414.

[S3] V. J. Inglezakis, S. G. Poulopoulos, H. Kazemian, Insights into the S-shaped sorption isotherms and their dimensionless forms, Microporous Mesoporous Mater. **2018**, 272 166–176, https://doi.org/10.1016/j.micromeso.2018.06.026.
